# Systemic Inflammation in Progressive Multiple Sclerosis Involves Follicular T-Helper, Th17- and Activated B-Cells and Correlates with Progression

**DOI:** 10.1371/journal.pone.0057820

**Published:** 2013-03-01

**Authors:** Jeppe Romme Christensen, Lars Börnsen, Rikke Ratzer, Fredrik Piehl, Mohsen Khademi, Tomas Olsson, Per Soelberg Sørensen, Finn Sellebjerg

**Affiliations:** 1 University of Copenhagen and Department of Neurology, Rigshospitalet, Copenhagen, Denmark; 2 Department of Clinical Neuroscience, Neuroimmunology Unit, Karolinska University Hospital, Stockholm, Sweden; University of Ottawa, Canada

## Abstract

Pathology studies of progressive multiple sclerosis (MS) indicate a major role of inflammation including Th17-cells and meningeal inflammation with ectopic lymphoid follicles, B-cells and plasma cells, the latter indicating a possible role of the newly identified subset of follicular T-helper (T_FH_) cells. Although previous studies reported increased systemic inflammation in progressive MS it remains unclear whether systemic inflammation contributes to disease progression and intrathecal inflammation. This study aimed to investigate systemic inflammation in progressive MS and its relationship with disease progression, using flow cytometry and gene expression analysis of CD4^+^ and CD8^+^T-cells, B-cells, monocytes and dendritic cells. Furthermore, gene expression of cerebrospinal fluid cells was studied. Flow cytometry studies revealed increased frequencies of ICOS^+^T_FH_-cells in peripheral blood from relapsing-remitting (RRMS) and secondary progressive (SPMS) MS patients. All MS subtypes had decreased frequencies of Th1 T_FH_-cells, while primary progressive (PPMS) MS patients had increased frequency of Th17 T_FH_-cells. The Th17-subset, interleukin-23-receptor^+^CD4^+^T-cells, was significantly increased in PPMS and SPMS. In the analysis of B-cells, we found a significant increase of plasmablasts and DC-SIGN^+^ and CD83^+^B-cells in SPMS. ICOS^+^T_FH_-cells and DC-SIGN^+^B-cells correlated with disease progression in SPMS patients. Gene expression analysis of peripheral blood cell subsets substantiated the flow cytometry findings by demonstrating increased expression of *IL21*, *IL21R* and *ICOS* in CD4^+^T-cells in progressive MS. Cerebrospinal fluid cells from RRMS and progressive MS (pooled SPMS and PPMS patients) had increased expression of T_FH_-cell and plasmablast markers. In conclusion, this study is the first to demonstrate the potential involvement of activated T_FH_-cells in MS. The increased frequencies of Th17-cells, activated T_FH_- and B-cells parallel findings from pathology studies which, along with the correlation between activated T_FH_- and B-cells and disease progression, suggest a pathogenic role of systemic inflammation in progressive MS. These observations may have implications for the treatment of progressive MS.

## Introduction

Progressive multiple sclerosis (MS) is characterized by steady progression of neurological disability without remission. Disability accumulation in progressive MS is severe and the time to development of a progressive disease course is the main determinant of the long-term prognosis [1], [2]. However, the pathogenetic understanding of disease progression is incomplete, and the development of treatments for progressive MS has so far been disappointing [3]. An unsolved question is to what extent disease progression is driven by inflammatory processes or axonal loss independent of inflammation. A low rate of relapses and gadolinium-enhancing lesions, pronounced atrophy and limited efficacy of treatment has supported a view where axonal loss independent of inflammation is thought to be the substrate for disease progression [4]. This view was challenged by recent pathology studies, which indicate that in progressive MS CNS inflammation is abundant and correlates with axonal damage and disease progression [5], [6]. Primary progressive (PPMS) and secondary (SPMS) progressive MS pathology is characterized by widespread diffuse inflammation with slowly expanding lesions, abundant cortical lesions, and lymphocyte infiltration and microglia activation in the normal appearing white matter (NAWM) [7]. The cellular density of infiltrates is generally lower than in acute lesions of RRMS, but progressive MS patients have higher numbers of B-cells and plasma cells in lesions, NAWM and meninges [5], [6]. Meningeal inflammation is pronounced in MS, and ectopic lymphoid follicle-like structures (ELFs) are observed in the meninges in progressive MS patients [6], [8]. ELFs are associated with more rapid disease progression, cortical lesions, meningeal and white matter inflammation, atrophy and neuronal loss [9], [10]. ELFs resemble lymphoid follicles with evidence of germinal center reactions, possibly facilitating the activation and differentiation of T- and B-cells within the CNS compartment [8]. The presence of ELFs is suggestive of the involvement of follicular T-helper (T_FH_) cells, a recently discovered T-cell subset, which is necessary for germinal center formation [11]. Additionally, monocytes and dendritic cells have been implicated in MS immunopathology [12]–[14].

Gene expression and immunohistochemistry studies of progressive MS brains have shown increased expression of pro-inflammatory cytokines, including interferon-gamma (IFNG), interleukin-17 (IL17), IL21, IL23 and tumor necrosis factor-alpha (TNFA) [15]–[19].

Thus, pathology studies have suggested CNS inflammation to be a key determinant for disease progression and axonal damage in progressive MS. The presence of ELFs and diffuse white matter inflammation with activated microglia could indicate a compartmentalization of inflammation, suggesting that CNS inflammation and disease progression in progressive MS could occur independent of systemic inflammation [4]. Several studies did, however, report increased activation of immune cells in peripheral blood from progressive MS patients including changes in surface phenotype [20]–[27] and expression of cytokines [28], [29], the most consistent being increased expression of IL12p40 and decreased expression of IL10 [25], [26], [29]–[32] indicative of a pro-inflammatory bias in progressive MS.

To date no systematic study of T-cells, B-cells, monocytes and dendritic cells has investigated systemic immune activation in progressive MS.

We hypothesized that patients with progressive MS have evidence of systemic immune activation, show signs of Th17- and T_FH_-cell inflammation and that this is associated with disease progression. Accordingly, we performed flow cytometry analysis on subsets of CD4^+^ and CD8^+^T-cells, B-cells, monocytes and dendritic cells, selected on the basis of previous progressive MS pathology and immunology studies, and subsequently related frequencies of significantly enriched subsets to disease activity. To substantiate findings from flow cytometry studies, we isolated peripheral blood mononuclear cell (PBMC) subsets and analyzed relevant targets by polymerase chain reaction (PCR). Finally, to relate the systemic immune activation with CNS inflammation, gene expression analysis of CSF cells and PBMCs was undertaken for the most important targets.

## Materials and Methods

### Patients and Healthy Controls

One cohort (Cohort A) including 20 relapsing-remitting MS (RRMS), 20 SPMS and 20 PPMS (all untreated) and 32 healthy controls (HC) was included for flow cytometry studies. Since the groups varied in age, we recruited healthy controls with an age and gender distribution that enabled us to make age- and gender-matched HC groups when a cell subset correlated with age. A second cohort (Cohort B) comprising 11 RRMS, 10 SPMS and 11 PPMS untreated patients and 12 HCs was used for gene expression studies on PBMC subsets. A third cohort (Cohort C) consisting of 45 SPMS patients, of whom 23 were untreated and 22 were treated with mitoxantrone, underwent studies of gene expression in whole blood (WB). Cohort A, B and C patients were recruited from the MS Clinic at Copenhagen University Hospital Rigshospitalet and had expanded disability status scale (EDSS) scores assigned by a neurologist (see Table 1).

**Table 1:** Demographic and clinical characteristics of MS patients, healthy controls and non-inflammatory neurological controls included in the studies.

	Group	N	Female/male	Age	MS duration	EDSS	EDSS change
**Flow cytometry**	HC	32	24/8	36 (29–46)			
	RRMS remission	20	14/6	38 (29–42)	6 (3–10)	2.3 (1.0–3.5)	0.5 (0.0–1.0)
	SPMS	20	11/9	48 (43–53)	14(11–21)	5.8 (4.8–6.5)	0.5 (0.5–1.0)
	PPMS	20	8/12	51 (45–54)	7 (4–9)	5.0 (4.0–6.0)	1.0 (0.5–2.0)
**Cell subsets**	HC	12	8/4	36 (29–45)			
	RRMS remission	10	7/3	34 (29–40)	6 (2–9)	1.3 (0.0–1.5)	0.0 (0.0-.5)
	SPMS	10	6/4	46 (33–52)	16 (10–19)	6.5 (6.0–6.5)	0.8 (0.5–1.0)
	PPMS	11	6/5	53 (44–55)	8 (3–10)	4.5 (4.0–6.0)	1.0 (0.0–1.5)
**Whole blood**	SPMS	23	12/11	50 (42–53)	16 (11–27)	5.5 (4.5–6.5)	0.5 (0.5–1.0)
	SPMS mitoxantrone	22	15/7	46 (42–54)	17 (14–24)	6.3 (5.9–6.5)	0.0 (0.0–0.4)
**CSF cell studies**	NIND	20	15/5	34 (31–39)			
	RRMS remission	10	8/2	44 (42–45)	8 (2–12)	2.8 (2.0–3.0)	
	RRMS relapse	10	6/4	37 (33–40)	3 (1–9)	3.3 (2.0–3.5)	
	SPMS	10	6/4	53(45–62)	20 (15–23)	5.5 (5.0–6.0)	
	PPMS	10	6/4	53 (44–63)	3 (2–8)	3.8 (3.5–4.5)	

Values for age, MS disease duration, EDSS and EDSS change are medians with interquartile ranges in brackets. EDSS change represents the change in EDSS the two previous years prior to blood sampling. Abbreviations: CSF = cerebrospinal fluid; EDSS = expanded disability status scale; HC = healthy control; MS = multiple sclerosis; NIND = non-inflammatory neurological disease; RRMS = relapsing-remitting multiple sclerosis; SPMS = secondary progressive multiple sclerosis; PPMS = primary progressive multiple sclerosis.

Finally, a cohort (Cohort D) consisting of 10 RRMS in clinical remission, 10 RRMS in clinical relapse, 10 SPMS and 10 PPMS untreated patients and 20 non-inflammatory neurological disease (NIND) patients (15 with sensory symptoms; 1 CADASIL; 1 carpal tunnel syndrome; 1 migraine with aura; 1 hypothyreosis with sensory disturbances; 1 vertigo) from Karolinska University Hospital was recruited for studies of gene expression in PBMCs and CSF cells.

The study protocol was approved by scientific ethics committee of the Capital Region of Denmark and the Karolinska Institute and written informed consent was obtained from the patients and healthy controls according to the Declaration of Helsinki.

### Samples

Blood was sampled in PAXgene (PreAnalytiX, Germany) and EDTA tubes. PBMCs were isolated using Lymphoprep (Axis-Shield, Norway). CSF was collected by lumbar puncture, immediately centrifuged and the CSF cell pellet snap-frozen.

### Flow Cytometry Studies

PBMCs were incubated with relevant combinations of flourochrome-conjugated monoclonal antibodies (Table S1A). WB was used for parallel assessment of absolute cell counts using TBNK reagent with TruCount tubes (BD Biosciences, USA). Data were acquired on a BD FACSCanto II flow cytometer and data analysis was done using BD FACSDiva Software (BD Biosciences, USA). In the development of the flow cytometry panel we systematically screened and titrated relevant antibodies for each antigen and subsequently assessed the most appropriate gating strategy for each subset. In the final data analysis, these strategies included the use of fluorescence-minus-one gating controls, isotype gating controls and visually based criteria.

### Gene Expression Studies

Subsets of CD4^+^T-cells, CD8^+^T-cells, B-cells, monocytes and dendritic cells (mean purity >92%) were isolated on an autoMACS separator using MACS cell separation kits (Miltenyi Biotec, Germany). RNA was extracted from 20,000–1,000,000 cells of the obtained subsets with PicoPure RNA Isolation Kit (Arcturus, USA). PAXgene Blood RNA Kit was used for PAXgene RNA extraction. CSF cell RNA was extracted using RNeasy Mini Kit (Qiagen, Germany). cDNA was synthesized with qScript cDNA SuperMix (Quanta BioSciences, USA) or iScript Supermix (Bio-Rad, USA). Real-time polymerase chain reactions (RT-PCR) were performed on a 7500 Fast or ViiA 7 Real-Time PCR System (Applied Biosystems, USA) using TaqMan Gene Expression Assays (Table S1B) and PerfeCTa FastMix (Quanta Bioscience, USA). Threshold cycle (CT) values were calculated using SDS software (Applied Biosystems, USA). The relative mRNA transcript expression was calculated using the 2^−ΔΔCT^ method. Reference genes were *CASC3* and *UBE2D2* in cell subset and WB studies and *UBC* for CSF cell and PBMC studies. Expression values were normalized to a HC PBMC cDNA pool resulting in a normalization ratio (NR). In the CSF cell studies we had a low RNA concentration, resulting in some samples being without amplification, in which case the NR was arbitrarily set to zero.

### Statistical Analysis

Statistical analysis was performed using PASW 19 software (IBM, USA). Patients with progressive MS were older than RRMS patients, and several cell-types change in frequency with age. Consequently we analyzed all flow cytometry variables from HCs for correlation with age using Spearman's rank correlation coefficient. Data were analyzed using ANCOVA with adjustment for age when a variable correlated significantly with age and otherwise ANOVA was used. A few variables were not normal distributed, and bootstrap procedures were applied to all ANOVA and ANCOVA tests. Since the analysis involved multiple comparisons we applied the false discovery rate (FDR) method to calculate q-values [33]. Q-values were considered significant when q< 0.05. Analysis for correlation between cell surface-phenotypes and disease progression was performed with Spearman's rank correlation coefficient. Gene expression data were analyzed with parametric statistics when appropriate and otherwise with non-parametric methods. Changes in the expression of mRNA were considered significant for p<0.05. The analysis of CSF gene expression data was restricted by the low RNA contents, and the resulting absence of amplification in some samples. This introduce variance and reduces the statistical power, and consequently we pooled the groups of RRMS in relapse and remission to a RRMS group and the group of SPMS and PPMS patients to a progressive group, resulting in three groups of each 20 individuals (NIND, RRMS and Progressive MS).

## Results

### Increased Frequencies of Circulating T_FH_- and Th17-cells in Progressive MS

**Figure 1:**
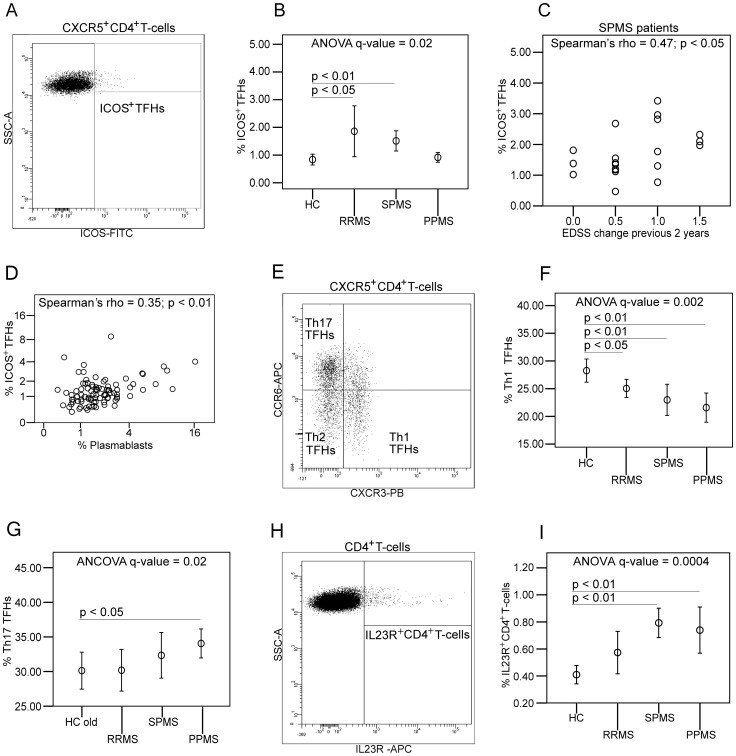
Flow cytometry studies on follicular T-helper cells and Th17-cells in peripheral blood from multiple sclerosis patients and healthy controls. Mean plots show mean percentages with error bars representing 95% confidence intervals. Dot plots show values for parameters tested in Spearman's rank correlation coefficient. Human blood follicular T-helper (T_FH_) cells are CXCR5^+^CD4^+^T-cells and as depicted on flow cytometry plot (A) ICOS^+^ T_FH_-cells were identified as ICOS^+^CXCR5^+^CD4^+^T-cells. (B) The frequency of ICOS^+^T_FH_-cells in blood is significantly increased in relapsing-remitting multiple sclerosis in clinical remission (RRMS) and in secondary progressive multiple sclerosis (SPMS) patients. (C) In the SPMS group the frequency of ICOS^+^T_FH_-cells significantly correlates with change in expanded disability status scale (EDSS) 2 years prior to sampling of blood. (D) A significant correlation between the frequencies of ICOS^+^T_FH_-cells and plasmablasts emphasize a known biological function of T_FH_-cells and that ICOS^+^T_FH_-cells are activated T_FH_-cells. (E) Flow cytometry plot showing the characterization of Th1 T_FH_-cells as CXCR3^+^CCR6^−^ T_FH_-cells, Th2 T_FH_-cells as CXCR3^−^CCR6^−^ T_FH_-cells and Th17 T_FH_-cells as CXCR3^−^CCR6^+^ T_FH_-cells. (F) Th1 T_FH_-cells are significantly decreased in all MS groups and (G) Th17 T_FH_-cells are significantly increased in primary progressive multiple sclerosis (PPMS) patients. (H) Th17-cells were identified as interleukin-23-receptor^+^ (IL23R^+^) CD4^+^T-cells and (I) there is significant increases of IL23R^+^CD4^+^T-cells in SPMS and PPMS patients.

We first conducted a detailed analysis of CD4^+^ and CD8^+^T-cells, B-cells, monocytes and dendritic cells in peripheral blood (Table S1A and S2A) Using FDR-correction for multiple corrections, significant differences between the patient groups were found mainly for CD4^+^T-cells. Examination of the phenotype of CXCR5^+^CD4^+^T-cells, presumably T_FH_-cells, revealed an increased frequency of an activated T_FH_-cell subset, ICOS^+^T_FH_-cells in RRMS and SPMS compared to HCs (Figure 1A–B).

Importantly, the frequency of ICOS^+^T_FH_-cells in SPMS correlated with EDSS change in the previous 2 years (Figure 1C). Frequencies of ICOS^+^T_FH_-cells and plasmablasts also correlated (Figure 1D), emphasizing the role of ICOS^+^T_FH_-cells for B-cell activation. CXCR3^+^, Th1-like T_FH_-cells, a subset lacking true ex vivo T_FH_ function [34], were reduced in frequency in all subtypes of MS compared to HCs, and in PPMS there was an increased frequency of CCR6^+^, Th17-like T_FH_-cells (Figure 1E, F, G).

Analysis of Th1- and Th17-phenotypes showed that SPMS and PPMS patients had an increased frequency of IL23-receptor (IL23R)^+^CD4^+^T-cells, presumably Th17-cells [35], in blood compared to HCs (Figure 1H–I).

The frequency of CD4^+^T-cells with a CD25^+^CD127^−^ regulatory T-cell (T_Reg_) phenotype was comparable in MS patients and controls [36]. There was, however, a decrease in the number of CD31^+^, recent thymic emigrant T_Reg_-cells in SPMS and PPMS patients, a finding which was paralleled by a decrease in CD31^+^CD4^+^T-cells in SPMS and PPMS patients (Table S2A).

### Antigen-presenting Cell Activation in Progressive MS

**Figure 2:**
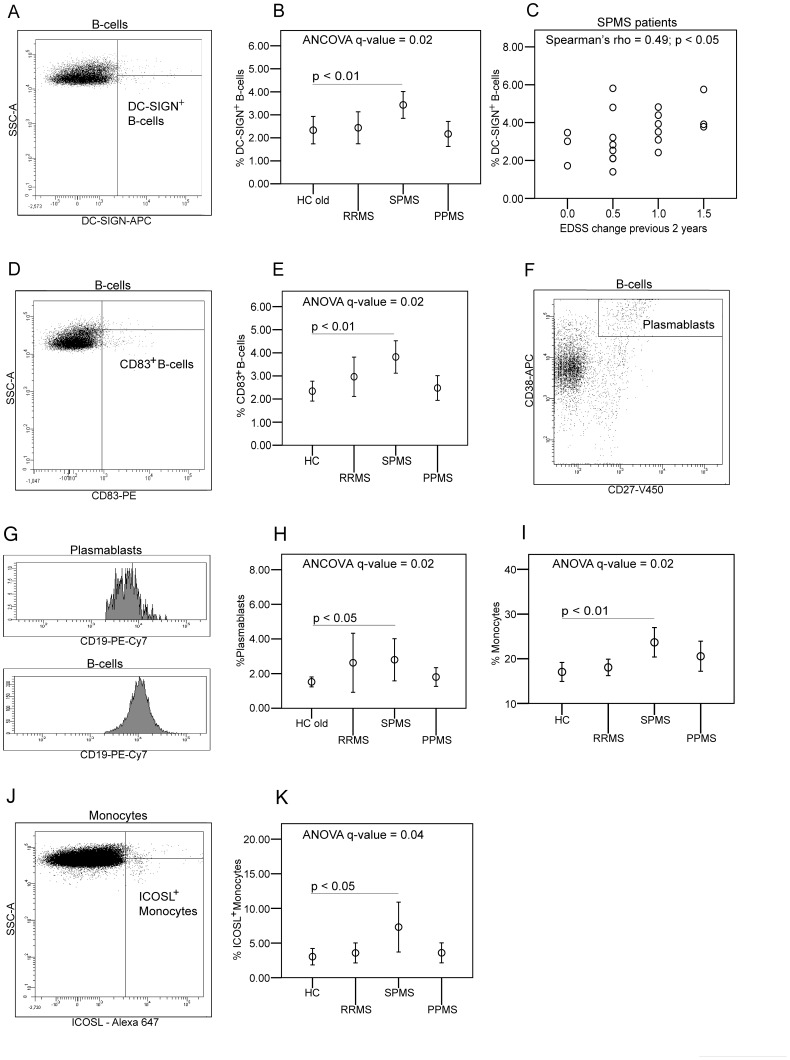
Flow cytometry studies on B-cell and monocytes in peripheral blood from multiple sclerosis patients and healthy controls. Mean plots show mean percentages with error bars representing 95% confidence intervals. Dot plots show values for parameters tested in Spearman's rank correlation coefficient. (A) Flow cytometry plot showing characterization of DC-SIGN^+^B-cells. (B) The frequency of DC-SIGN^+^B-cells is significantly increased in secondary progressive multiple sclerosis (SPMS) patients as compared to healthy controls (HC). (C) The frequency of DC-SIGN^+^B-cells correlates significantly with change in expanded disability status scale (EDSS) 2 years prior to sampling of blood in the SPMS group. (D) CD83^+^B-cells were identified as shown on flow cytometry plot and (E) the frequency is significantly increased in SPMS patients compared to HCs. (F) Plasmablasts were identified as CD27^High^CD38^High^B-cells and (G) have lower CD19 expression than total B-cells, consistent with a plasmablast phenotype. (H) Plasmablasts are increased in SPMS patients compared to age-matched healthy controls (HC old). (I) Monocyte frequency is significantly increased in SPMS patients compared to HCs. (J) Identification of ICOS-ligand^+^ (ICOSL) monocytes is shown on flow cytometry plot and the frequency is increased in SPMS patients compared to HCs (K).

In our flow cytometry analyses we included a detailed phenotyping of B-cells, monocytes, myeloid and plasmacytoid dendritic cells (Table S1 and S2B–C), all cell types with known antigen-presenting cell (APC) function. We found higher frequencies of DC-SIGN^+^B-cells and CD83^+^B-cells in SPMS than in HCs and DC-SIGN^+^B-cells frequency correlated with disease progression in SPMS (Figure 2A, B, C, D, E). Of note both subsets correlated significantly with IL23R^+^CD4^+^T-cells and ICOS^+^T_FH_-cells (Figure S1), indicating an association between these T- and B-cell subsets. Plasmablasts were identified as CD27^High^CD38^High^B-cells and were increased in SPMS patients compared to HCs (Figure 2F, G, H). We also observed an increased percentage of monocytes and monocytes expressing ICOS-ligand (ICOSL) in SPMS (Figure 2J, K), whereas other differences observed for B-cells, monocytes and dendritic cells were not statistically significant after FDR-correction for multiple comparisons.

### Gene Expression in CD4^+^T-cells, CD8^+^T-cells, B-cells, Monocytes and Dendritic Cells

**Figure 3:**
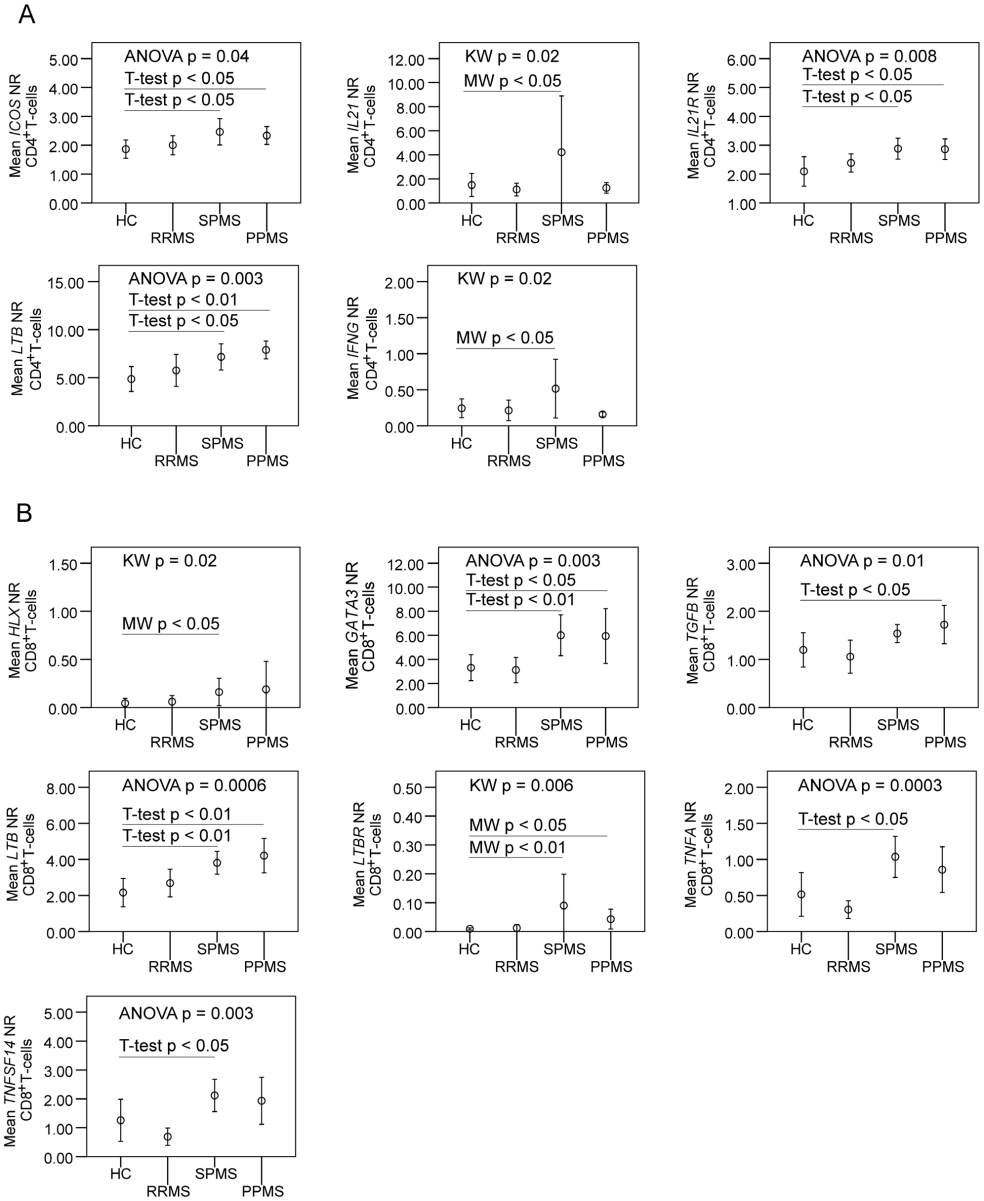
Gene expression studies of peripheral blood CD4^+^ and CD8^+^T-cells from multiple sclerosis patients and healthy controls. Mean plots show mean normalization ratios (NR) with error bars representing 95% confidence intervals. Parametric (ANOVA and post-hoc T-tests) or non-parametric (Kruskal-Wallis (KW) and Mann-Whitney (MW)) statistic tests were applied when suitable. (A) Gene expression analysis in CD4^+^T-cells shows increased expression of *ICOS*, *IL21R* and *LTB* in secondary progressive (SPMS) and primary progressive (PPMS) multiple sclerosis patients as compared to healthy controls (HC) and increased expression of *IL21* and *IFNG* in SPMS patients compared to HCs. (B) Gene expression analysis in CD8^+^T-cells shows increased expression of *GATA3*, *LTB* and *LTBR* in SPMS and PPMS compared to HCs. *HLX*, *TGFB*, *TNFA* and *TNFSF14* were increased in SPMS compared to HCs.

To substantiate the findings in the flow cytometry studies, we analyzed gene expression in purified CD4^+^T-cells, CD8^+^T-cells, B-cells, monocytes and dendritic cells (Table S3). Expression of *ICOS*, *IL21* and *IL21R* (IL21-receptor) in CD4^+^T-cells from SPMS patients was increased, which is suggestive of increased activation of T_FH_-cells in SPMS (Figure 3A). In addition, SPMS patients had increased expression of *IFNG* in CD4^+^T-cells and of *LTB* (lymphotoxin-beta) in CD4^+^ and CD8^+^T-cells and of *TNFA*, *LTBR* (LTB-receptor), *TNFSF14* (LIGHT), and the transcription factors *HLX* and *GATA3* in CD8^+^T-cells. In PPMS patients there was higher expression of *ICOS*, *IL21R* and *LTB* in CD4^+^T-cells and of *LTB*, *LTBR*, *TGFB1* (transforming growth factor-beta) and *GATA3* in CD8^+^T-cells.

**Figure 4:**
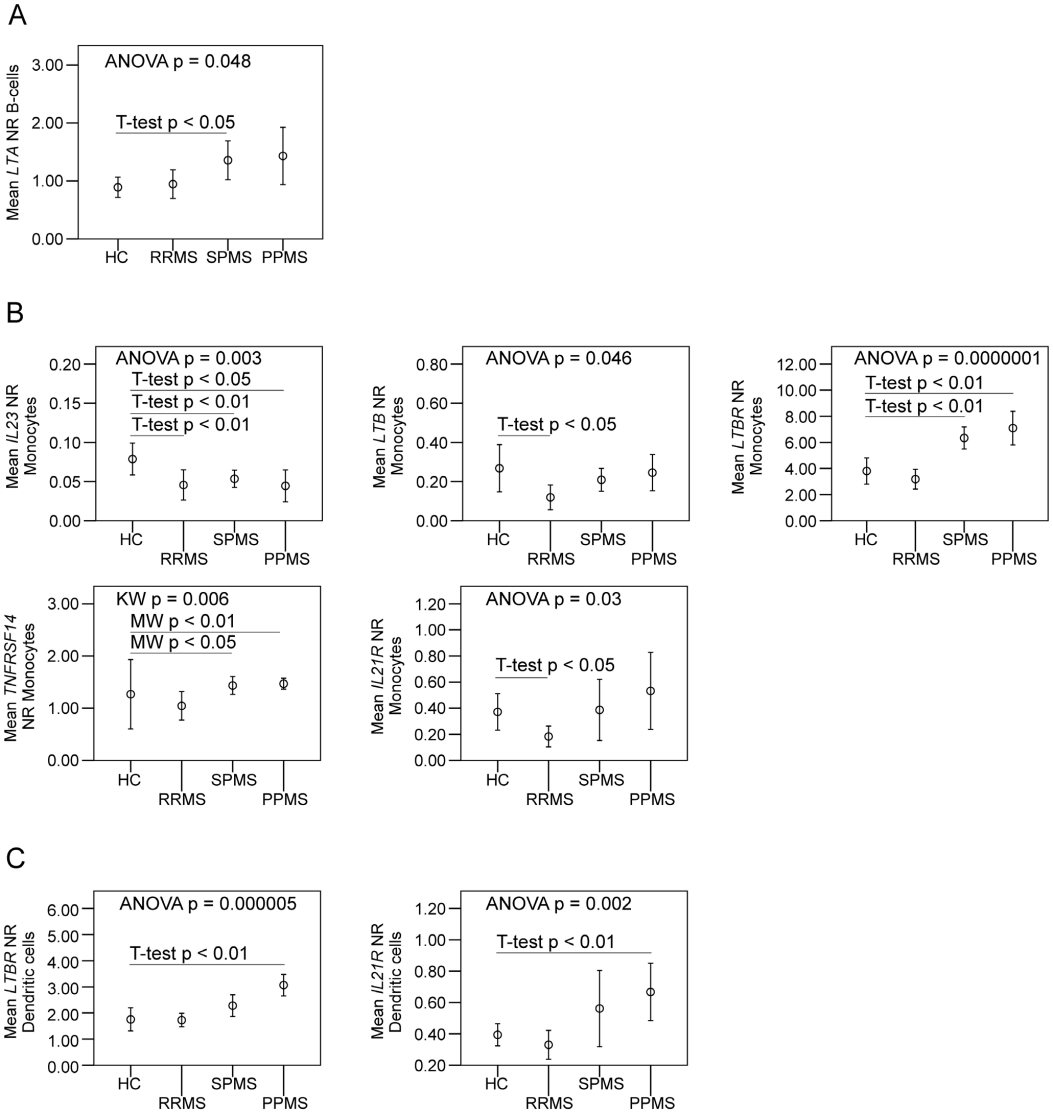
Gene expression studies of peripheral blood B-cells, monocytes and dendritic cells from multiple sclerosis patients and healthy controls. Mean plots show mean normalization ratios (NR) with error bars representing 95% confidence intervals. Parametric (ANOVA and post-hoc T-tests) or non-parametric (Kruskal-Wallis (KW) and Mann-Whitney (MW)) statistic tests were applied when suitable. (A) B-cells have increased expression of *LTA* in secondary progressive multiple sclerosis (SPMS) patients as compared to healthy controls (HC). (B) Gene expression in monocytes, showing decreased expression of *IL23* in relapsing-remitting multiple sclerosis in remission (RRMS), SPMS and primary progressive multiple sclerosis (PPMS) compared to HCs and decreased *LTB* and *IL21R* expression in monocytes in RRMS patients compared to HCs. Increased expression of *LTBR* and *TNFRSF14* in monocytes is observed for SPMS and PPMS patients. (C) Dendritic cells have increased expression of *LTBR* and *IL21R* in PPMS patients compared to HCs.

Observed gene expression changes in APCs corroborated the findings in T-cells (Figure 4). B-cells from SPMS patients had increased expression of *LTA* (lymphotoxin-alpha), monocytes from SPMS and PPMS patients had increased expression of *LTBR* and *TNFRSF14* (HVEM), and dendritic cells from patients with PPMS had increased expression of *LTBR* and *IL21R*.

### Cerebrospinal Fluid Cell Expression of T_FH_-cell and Plasmablast Genes

The finding of increased frequencies of ICOS^+^T_FH_-cells in SPMS patients and TH17-like T_FH_-cells in PPMS patients prompted us to analyze measures of T_FH_-cell activation in samples of CSF cells. We found significantly increased expression of *ICOS* and *IGJ* (immunoglobulin J chain) in progressive MS and RRMS patients, and increased *LTA* expression in CSF cells from RRMS patients (Table 2). In spite of being a low abundance transcript we were able to detect *IL21* expression in 60% of progressive MS samples and 10% of NIND and RRMS samples, and there was trend (p = 0.06) towards increased *IL21* expression in CSF cells from progressive MS patients. In the PBMCs we could not reproduce the finding of increased *IL21* expression in CD4^+^T-cells in SPMS patients, which is likely explained by the pooling of the groups and by the low expression level with *IL21* detectable in only 53% of the PBMC samples.

**Table 2:** Cerebrospinal fluid cell and peripheral blood mononuclear cell gene expression data.

		NIND				RRMS				Progressive MS				
		N	Mean	SE		N	Mean	SE		N	Mean	SE		Kruskal-Wallis∼/ ANOVA^∧^ p-value
***ICOS PBMC NR***		20	0.41	0.03		20	0.44	0.04		20	0.42	0.04		0.89^∧^
***ICOS CSF NR***		20	0.54	0.12		20	**0.94^∧^**	0.14		20	**0.89^∧^**	0.09		**0.04^∧^**
														
***IGJ PBMC NR***		20	1.53	0.85		20	1.05	0.19		20	0.72	0.08		0.45∼
***IGJ CSF NR***		20	0.13	0.03		20	**1.57≈**	0.25		20	**1.43≈**	0.35		**0.000002∼**
														
***IFNG*** ** PBMC NR**		20	0.31	0.05		20	0.23	0.04		20	0.33	0.07		0.48∼
***IFNG*** ** CSF NR**		20	0.11	0.06		20	0.09	0.07		20	0.11	0.06		0.53∼
														
***IL21 PBMC NR***		20	0.74	0.2		20	0.72	0.22		20	0.71	0.18		0.98∼
***IL21 CSF NR***		20	0.29	0.24		20	0.37	0.37		20	1.96	0.85		0.06∼
														
***LTA*** ** PBMC NR**		20	0.25	0.03		20	0.36	0.04		20	0.28	0.03		0.15∼
***LTA*** ** CSF NR**		20	0.13	0.08		20	**0.48∼**	0.13		20	0.19	0.06		**0.04∼**
														
***LTB*** ** PBMC NR**		20	1.63	0.13		20	1.78	0.18		20	1.98	0.18		0.30^∧^
***LTB*** ** CSF NR**		20	2.99	0.29		20	3.14	0.34		20	2.73	0.21		0.59^∧^

Gene expression data cerebrospinal fluid cell (CSF) and peripheral blood mononuclear cell (PBMC) are expressed as normalization ratio (NR). Values are expressed as means with standard error (SE). Parametric (ANOVA and T-test) and non-parametric analyses (Kruskal-Wallis and Mann-Whitney) were used when appropriate. Post-hoc analyses was done when ANOVA or Kruskal-Wallis tests were significant (p = 0.05) and values for post-hoc tests are indicated (T-tests: ^∧^ p<0.05 and Mann-Whitney ∼ p<0.05; ≈ p<0.01).Relapsing-remitting multiple sclerosis (RRMS) group consists of 10 patients in clinical remission and 10 patients with clinical relapse. Progressive multiple sclerosis (MS) consists of 10 secondary progressive and 10 primary progressive MS patients. Abbreviations: Non-inflammatory neurological disease = NIND.

### Mitoxantrone Treated SPMS Patients have Reduced Expression of *IL21*

**Figure 5:**
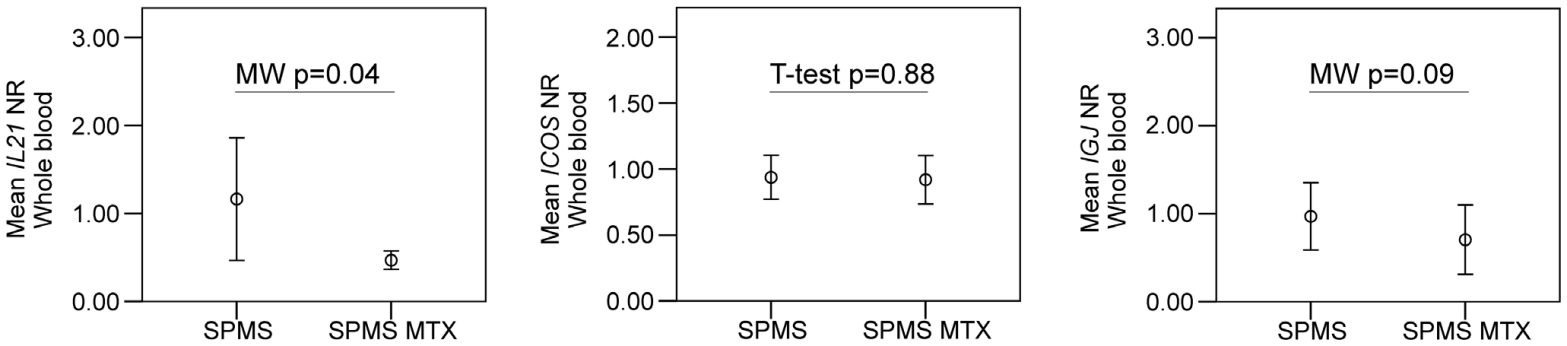
Gene expression studies of whole blood in secondary progressive multiple sclerosis with and without mitoxantrone treatment. Mean plots show mean normalization ratios (NR) with error bars representing 95% confidence intervals. Parametric (T-test) or non-parametric (Mann-Whitney (MW)) statistic tests were applied when suitable. Comparing untreated secondary progressive multiple sclerosis (SPMS) patients to SPMS patient treated with mitoxantrone (SPMS MTX) whole blood expression of *IL21* significantly decreased while no significant changes were observed for *ICOS* and *IGJ*.

Finally we sought to determine if immunosuppressive treatment with mitoxantrone is associated with alterations in the expression of genes associated with T_FH_ activation in blood cells. As shown (Figure 5), SPMS patients treated with mitoxantrone had significantly lower *IL21* expression, whereas there were no significant differences in *ICOS* and *IGJ* gene expression.

## Discussion

Previous studies have indicated the presence of systemic and intrathecal inflammation in progressive MS, but it is unclear to what extent systemic inflammation mirrors intrathecal inflammation and whether systemic inflammation contributes to intrathecal inflammation and disease progression. The present study is the first to report prominent T_FH_-, Th17- and B-cell activation in blood from patients with progressive MS, findings which parallel recent pathology studies. T_FH_- and B-cell activation correlated with disease progression in SPMS and T_FH_ activation marker *IL21* was decreased in SPMS patients treated with mitoxantrone. Further we found increased expression of genes associated with T_FH_- and B-cell activation in CSF cells from RRMS and progressive MS patients. These findings emphasize an association between the systemic immune compartment and disease progression in progressive MS.

We initially studied immune activation using a broad flow cytometry characterization of blood cell subsets, correcting for multiple comparisons in the statistical analysis by the FDR-method and correcting for age effects when appropriate. In accordance with a previous study we found similar frequencies of CXCR5^+^CD4^+^T-cells in MS and HCs [37]. ICOS^+^T_FH_-cells are activated T_FH_-cells with a phenotype similar to germinal center T_FH_-cells, which are capable of activating B-cells and produce the effector cytokine IL21 [11]. ICOS^+^T_FH_-cell frequency was increased in RRMS and SPMS patients, and correlated with disease progression in SPMS patients. Others have phenotyped human T_FH_-cells according to CCR6 and CXCR3 expression, resulting in division into Th1, Th2 and Th17-like T_FH_-cell subsets, of which only the latter two are capable of inducing B-cells to produce immunoglobulin via IL21 [34]. We found that all subtypes of MS patients have a lower percentage of Th1 T_FH_-cells, whereas PPMS patients had an increased percentage of Th17 T_FH_-cells, consistent with a skewing towards activated T_FH_-cells. We also found a decrease of CD31+ *recent thymic emigrant* cells in patients with PPMS and SPMS, as previously reported [38]. Other previous studies found increased CD40-ligand, CCR2 and CCR5 expression on CD4^+^ and CD8^+^T-cells in SPMS [23], [39]. These findings were not confirmed in the present study.

Expression of *IL21R* and *ICOS* was increased in purified CD4^+^T-cells from SPMS and PPMS patients while SPMS patients also had increased *IL21* expression. In CSF cells from patients with progressive MS *ICOS* expression was increased. These molecules all have known effector T_FH_ functions and the observation is thus compatible with T_FH_-cell activation [11], [40]. Furthermore, expression of *IL21* mRNA was lower in blood cells from SPMS patients treated with mitoxantrone, suggesting that inhibition of T_FH_ function may be an effect of mitoxantrone in SPMS. However, it is worth noting that IL21 and ICOS are also expressed by Th17-cells and a CCR9^+^CD4^+^T-cells, a subset with resemblance to T_FH_-cells [41], [42]. Indeed, animal and human studies have described overlapping features of Th17- and T_FH_-cell biology [11], [43], [44], and recently it was shown that Th17-cell-induced EAE mice develop ELFs, possibly caused by the differentiation of Th17-cells to T_FH_-cells [45]. Thus, the finding of increased *IL21* and *ICOS*, is not definite proof of T_FH_ -activity but could also be a result of Th17-activation. Detection of the Th17 signature cytokine IL17 in purified CD4^+^T-cells was limited to few individuals and therefore we could not substantiate whether the increase in IL23R^+^CD4^+^T-cells in SPMS and PPMS reflects a true increase in Th17 activity or a potential for increased Th17 effector function in progressive MS.

The flow cytometry finding of skewing towards activation of T_FH_-cells in all MS subtypes corresponds to results of histopathology studies which have demonstrated the presence of IL21R^+^ and IL21^+^CD4^+^T-cells in both active and chronic lesions [19] and meningeal inflammation with closely associated T- and B-cells in all MS subtypes [10], [46]. Furthermore, the findings are consistent with the presence of ELFs in some SPMS cases. In this context, it has been suggested that ELFs represents an end-stage of meningeal inflammation, which fits well with the long disease duration of SPMS cases [46]. Taken together these findings could indicate that activation of T_FH_-cells is a feature of all MS subtypes.

The extent to which the observed changes in T_FH_ - and Th17-phenotypes and recent thymic emigrants are associated with functional changes in immune reactivity must be established in functional studies, but we hypothesize that the changes in T_FH_ phenotype may reflect an increased potential for T_FH_ interactions with B-cells and that the decrease in CD31^+^T-cells may reflect an increased memory and effector T-cell pool in patients with progressive MS.

A second main finding is the confirmation of aberrant B-cell activation in MS, where CD80^+^B-cells and plasmablasts are increased in the CSF and correlate with intrathecal immunoglobulin G production [47], [48]. Thus, we observed increased percentages of plasmablasts, DC-SIGN^+^B-cells and CD83^+^B-cells in blood from SPMS, and DC-SIGN^+^B-cells and disease progression correlated in SPMS. DC-SIGN^+^B-cells and CD83^+^B-cells also correlated positively with IL23R^+^CD4^+^T-cells and ICOS^+^T_FH_-cells, consistent with a previously described relationship between activated T- and B-cell subsets [49], [50]. In fact, B-cells are known to be important for development and sustaining of T_FH_- and Th17-responses [11], [51], [52] No studies have described DC-SIGN^+^B-cells and CD83^+^B-cells in MS, but DC-SIGN^+^B-cells have been shown to be activated B-cells in humans [53], and in animal studies CD83^+^B-cells are involved in antigen-specific T- and B-cell interactions [54]. Interestingly, several studies have used DC-SIGN and CD83 as markers for dendritic cells and found increased frequency of cells expressing DC-SIGN and CD83 in MS brains [12]–[14].

Among antigen-presenting cells we further found increased frequencies of monocytes and ICOSL^+^monocytes in SPMS patients, and in gene expression studies we also found increased expression of several TNF and TNF-receptor superfamily molecules in monocytes and dendritic cells from MS patients. In general, however, the changes observed in APCs were less prominent than those observed in T-cells.

Our experimental approach is well suited for analyzing the *in vivo* activation stage of immune cells since there is minimal manipulation of the cells prior to the analysis. Studies relying on *in vitro* activation may, however, reveal differences in the potential for activation which analyses of freshly isolated cells might neglect. The most consistent findings from *in vitro* studies in progressive MS have been increased IL12p40 and IFNG and decreased IL10 production by stimulated PBMCs [25], [30]–[32], [55], [56] in SPMS. IL12p40 is translated from *IL12B* mRNA, which in our study was expressed only in B-cells and not in monocytes or dendritic cells. Therefore, it appears that the study of *in vitro* stimulated monocytes and dendritic cells is needed to identify some of the changes in APC activation in patients with MS.

The formation of ELFs is critically dependent on the TNF and TNF-receptor superfamily molecules LTA, LTB, LTBR, TNFSF14 and TNFRSF14 [26], [57], [58]. We found increased expression of at least one of these transcripts in all the subsets of blood cells in progressive MS, indicating an involvement of the cytokines which likely could be linked to the development of ELFs in the meninges in progressive MS patients.

Collectively, the results of the present study suggest a central role of T_FH_ -cells along with Th17-cells and activated B-cell subsets in the pathogenesis of progressive MS, and indicate that systemic inflammation is associated with disease progression. Consequently, the findings support that treatments targeting T_FH_-, Th17- or B-cells may be efficacious in the treatment of progressive MS [49], [59]. Of notice, B-cell depleted mice are also deficient in T_FH_-cells [11], [52]. Indeed, the findings of recent clinical trials with B-cell-depleting monoclonal antibodies indicate that B cell depletion may not only be efficacious in RRMS, but also in a subgroup of PPMS patients characterized by contrast-enhancing lesions or younger age [60].

## Supporting Information

Table S1 **Lists of antibodies and TaqMan gene expression assays used in the studies.**
(DOCX)

Table S2 **Flow cytometry data from healthy controls (HC), relapsing-remitting multiple sclerosis (RRMS), secondary progressive multiple sclerosis (SPMS) and primary progressive multiple sclerosis (PPMS) patients.**
(DOCX)

Table S3 **Gene expression data from CD4^+^T–cells, CD8^+^T–cells, B–cells, monocytes and dendritic cells.**
(DOCX)

Figure S1 ****
(TIF)
