# Correction: Systemic Inflammation in Progressive Multiple Sclerosis Involves Follicular T-Helper, Th17- and Activated B-Cells and Correlates with Progression

**DOI:** 10.1371/annotation/b4e623eb-4950-48d9-8d85-8d70426d95a3

**Published:** 2013-03-05

**Authors:** Jeppe Romme Christensen, Lars Börnsen, Rikke Ratzer, Fredrik Piehl, Mohsen Khademi, Tomas Olsson, Per Soelberg Sørensen, Finn Sellebjerg

Dr. Jeppe Romme Christensen's last name is Romme Christensen. The correct citation is: Romme Christensen J, Börnsen L, Ratzer R, Piehl F, Khademi M, et al. (2013) Systemic Inflammation in Progressive Multiple Sclerosis Involves Follicular T-Helper, Th17- and Activated B-Cells and Correlates with Progression. PLoS ONE 8(3): e57820. doi:10.1371/journal.pone.0057820

