# Supplementary material for: Systemic Inflammation in Progressive Multiple Sclerosis Involves Follicular T-Helper, Th17- and Activated B-Cells and Correlates with Progression
Source: PLoS One. 2013 Mar 1;8(3):e57820. doi: 10.1371/journal.pone.0057820 (PMC3585852; doi:10.1371/journal.pone.0057820)
Supplement: Table S1 — Lists of antibodies and TaqMan gene expression assays used in the studies. (DOCX) [file pone.0057820.s001.docx]

**Table S1: Lists of antibodies and TaqMan gene expression assays used in the studies.** Table S1A lists the combinations of antibodies used along with corresponding flourochrome conjugates, clone-names and suppliers. Table S1B lists the TaqMan Gene Expression Assays used in gene expression studies of peripheral blood mononuclear cell (PBMC) subsets, cerebrospinal fluid (CSF) cells and PBMCs and whole blood.

| **Table S1A** | | |  |  |  |  |  |  |  |  |  |  |  |  |  |  |  |  |
| --- | --- | --- | --- | --- | --- | --- | --- | --- | --- | --- | --- | --- | --- | --- | --- | --- | --- | --- |
|  |  |  |  |  |  |  |  |  |  |  |  |  |  |  |  |  |  |  |
| **Antigen presenting cells (APC)** | | | | | | | | |  | **T-cells** | | | | | | | | |
|  |  |  |  |  |  |  |  |  |  |  |  |  |  |  |  |  |  |  |
|  |  | **Antigen** |  | **Conjugate** |  | **Clone** |  | **Supplier** |  |  |  | **Antigen** |  | **Conjugate** |  | **Clone** |  | **Supplier** |
| **All APC tubes** |  | CD1c (BDCA-1) |  | PerCP-Cy5.5 |  | L161 |  | Biolegend |  | **All T-cell tubes** |  | CD3 |  | APC-Cy7 |  | HIT3a |  | Biolegend |
|  |  | CD303 (BDCA-2) |  | FITC |  | AC144 |  | Miltenyi |  |  |  | CD4 |  | PE-Cy7 |  | RPA-T4 |  | Biolegend |
|  |  | CD19 |  | PE-Cy7 |  | HIB19 |  | Biolegend |  |  |  | CD8 |  | eFluor 605NC |  | RPA-T8 |  | eBioscience |
|  |  | CD14 |  | Qdot 605 |  | TüK4 |  | Invitrogen |  |  |  |  |  |  |  |  |  |  |
|  |  |  |  |  |  |  |  |  |  | **T-cell tube 1** |  | CD161 |  | PerCP-Cy5.5 |  | HP-3G10 |  | Biolegend |
| **APC Tube 1** |  | CD209 (DC-SIGN) |  | APC |  | 9E9A8 |  | Biolegend |  |  |  | IL12RB2 |  | APC |  | 305719 |  | R&D systems |
|  |  | CD83 |  | PE |  | HB15e |  | Biolegend |  |  |  | IL23R |  | FITC |  | 218213 |  | R&D systems |
|  |  | CCR7 |  | PB |  | TG8/CCR7 |  | Biolegend |  |  |  | CCR2 |  | PE |  | 48607 |  | R&D systems |
|  |  |  |  |  |  |  |  |  |  |  |  |  |  |  |  |  |  |  |
| **APC Tube 2** |  | CD38 |  | APC |  | HIT2 |  | Biolegend |  | **T-cell tube 2** |  | CXCR5 |  | PerCP-Cy5.5 |  | TG2/CXCR5 |  | Biolegend |
|  |  | CD138 |  | PE |  | MI15 |  | Biolegend |  |  |  | CCR6 |  | APC |  | 11A9 |  | BD Biosciences |
|  |  | CD27 |  | V450 |  | M-T271 |  | BD Biosciences |  |  |  | CCR5 |  | FITC |  | 45531 |  | R&D systems |
|  |  |  |  |  |  |  |  |  |  |  |  | CXCR3 |  | PB |  | TG1/CXCR3 |  | Biolegend |
| **APC Tube 3** |  | CD40 |  | APC |  | HB14 |  | Biolegend |  |  |  |  |  |  |  |  |  |  |
|  |  | CD70 |  | PE |  | Ki-24 |  | BD Biosciences |  | **T-cell tube 3** |  | CXCR5 |  | PerCP-Cy5.5 |  | TG2/CXCR5 |  | Biolegend |
|  |  | CD86 |  | PB |  | IT2.2 |  | Biolegend |  |  |  | CD134 (OX40) |  | APC |  | 443318 |  | R&D systems |
|  |  |  |  |  |  |  |  |  |  |  |  | CD278 (ICOS) |  | FITC |  | ISA-3 |  | eBioscience |
| **APC Tube 4** |  | CD275 (ICOSL) |  | Alexa 647 |  | MIH11 |  | AbD Serotec |  |  |  | CD279 (PD-1) |  | PE |  | EH12.2H7 |  | Biolegend |
|  |  | CD252 (OX40L) |  | PE |  | 11C3.1 |  | Biolegend |  |  |  | CD154 (CD40L) |  | PB |  | 24-31 |  | Biolegend |
|  |  | CD80 |  | V450 |  | L307.4 |  | BD Biosciences |  |  |  |  |  |  |  |  |  |  |
|  |  |  |  |  |  |  |  |  |  | **T-cell tube 4** |  | CD25 |  | PerCP-Cy5.5 |  | BC96 |  | Biolegend |
| **APC Tube 5** |  | IL15 |  | PE |  | IC2471P |  | R&D systems |  |  |  | CD127 |  | FITC |  | eBioRDR5 |  | eBioscience |
|  |  |  |  |  |  |  |  |  |  |  |  | CD31 |  | PB |  | WM59 |  | Biolegend |
| **APC tube 6** |  | CD274 (PDL1) |  | APC |  | 29E.2A3 |  | Biolegend |  |  |  |  |  |  |  |  |  |  |
|  |  | CD273 (PDL2) |  | PE |  | 176611 |  | R&D systems |  |  |  |  |  |  |  |  |  |  |

| **Table S1B** | | | |  |  |  |  |
| --- | --- | --- | --- | --- | --- | --- | --- |
|  |  |  |  |  |  |  |  |
| **Gene** | **Assay number** |  | **PBMC subset studies** |  | **CSF cells and PBMC studies** |  | **Whole blood studies** |
| *UBE2D2* | Hs00366152_m1 |  | • |  |  |  | • |
| *CASC3* | Hs00904832_m1 |  | • |  |  |  | • |
| *UBC* | Hs00824723_m1 |  |  |  | • |  |  |
| *HLX1* | Hs00172035_m1 |  | • |  |  |  |  |
| *TBX21* | Hs00203436_m1 |  | • |  |  |  |  |
| *GATA3* | Hs00231122_m1 |  | • |  |  |  |  |
| *RORC* | Hs01076112_m1 |  | • |  |  |  |  |
| *IFNG* | Hs99999041_m1 |  | • |  | • |  |  |
| *IGJ* | Hs00376160_m1 |  | • |  | • |  | • |
| *IL4* | Hs00174122_m1 |  | • |  |  |  |  |
| *IL6* | Hs00174131_m1 |  | • |  |  |  |  |
| *IL10* | Hs00961622_m1 |  | • |  |  |  |  |
| *IL12A* | Hs00168405_m1 |  | • |  |  |  |  |
| *IL12B* | Hs00233688_m1 |  | • |  |  |  |  |
| *IL17A* | Hs00174383_m1 |  | • |  |  |  |  |
| *IL1B* | Hs00174097_m1 |  | • |  |  |  |  |
| *IL21* | Hs00222327_m1 |  | • |  | • |  | • |
| *IL23* | Hs00372324_m1 |  | • |  |  |  |  |
| *TGFB* | Hs99999918_m1 |  | • |  |  |  |  |
| *TNFA* | Hs00174128_m1 |  | • |  |  |  |  |
| *ICOS* | Hs00359999_m1 |  | • |  | • |  | • |
| *IL21R* | Hs00986138_m1 |  | • |  |  |  |  |
| *IL23R* | Hs00332759_m1 |  | • |  |  |  |  |
| *LTA* | Hs00236874_m1 |  | • |  | • |  |  |
| *LTB* | Hs00242739_m1 |  | • |  | • |  |  |
| *LTBR* | Hs01101194_m1 |  | • |  |  |  |  |
| *TNFRSF14* | Hs00998604_m1 |  | • |  |  |  |  |
| *TNFSF14* | Hs00542477_m1 |  | • |  |  |  |  |
