# Supplementary material for: Systemic Inflammation in Progressive Multiple Sclerosis Involves Follicular T-Helper, Th17- and Activated B-Cells and Correlates with Progression
Source: PLoS One. 2013 Mar 1;8(3):e57820. doi: 10.1371/journal.pone.0057820 (PMC3585852; doi:10.1371/journal.pone.0057820)
Supplement: Table S2 — Flow cytometry data from healthy controls (HC), relapsing-remitting multiple sclerosis (RRMS), secondary progressive multiple sclerosis (SPMS) and primary progressive multiple sclerosis (PPMS) patients. (DOCX) [file pone.0057820.s002.docx]

**Table S2: Flow cytometry data from healthy controls (HC), relapsing-remitting multiple sclerosis (RRMS), secondary progressive multiple sclerosis (SPMS) and primary progressive multiple sclerosis (PPMS) patients.** (A) Absolute cell counts and frequencies of CD4^+^ and CD8^+^T-cells and their surface phenotypes are shown. CXCR5^+^CD4^+^T-cells are follicular T-helper (T_FH_) cells and Th1 T_FH_-cells were characterized as CXCR3^+^CCR6^-^ T_FH_-cells, Th2 T_FH_-cells as CXCR3^-^CCR6^-^ T_FH_-cells and Th17 T_FH_-cells as CXCR3^-^CCR6^+^ T_FH_-cells. CD4^+^regulatory T-cells (CD4 T_Reg_-cells) were characterized as CD25^+^CD127^Low^ CD4^+^T-cells. (B) Absolute cell counts and frequencies of B-cells and monocytes and their surface phenotypes are shown. Plasmablasts were characterized as CD27^High^CD38^High^B-cells. (C) Frequencies of myeloid and plasmacytoid dendritic cells and their surface phenotypes. Spearman’s correlation coefficient (R) was used for analysis of dependence between age and each cell variable (Corr. with age) in the HC group; *= p≤0.05 and **= p≤0.01. ANOVA tests were used for test of significance, except when significant correlation with age in HCs was observed in which case ANCOVA with adjustment for age was used. False discovery rate (FDR) was used to calculate q-values for correction for multiple significance tests. Post-hoc T-tests were performed for variables significant after FDR-correction, and testing was done against HCs as whole group (⁰= p≤0.05 and ⁰⁰= p≤0.01), or in the case of significant correlation with age, an age-matched group consisting of 20 HCs with appropriate age selected from the HC-group (old †= p≤0.05 and ‡= p≤0.01). Significant values are in bold.

Abbreviations: HC = healthy control; NS = non-significant; PPMS = primary progressive multiple sclerosis RRMS = relapsing-remitting multiple sclerosis; SPMS = secondary progressive multiple sclerosis; T_FH_ = follicular T-helper; T_Reg_ = Regulatory T-helper cell.

| **Table S2A** | | |  |  |  |  |  |  |  |  |  |  |  |  |  |  |  |  |  |  |  |  |  |  |  |  |  |  |  |  |  |  |  |
| --- | --- | --- | --- | --- | --- | --- | --- | --- | --- | --- | --- | --- | --- | --- | --- | --- | --- | --- | --- | --- | --- | --- | --- | --- | --- | --- | --- | --- | --- | --- | --- | --- | --- |
|  |  |  |  |  |  |  |  |  |  |  |  |  |  |  |  |  |  |  |  |  |  |  |  |  |  |  |  |  |  |  |  |  |  |
| **CD4^+^T-cells** | | | | | | | | | | | | | | | | |  | **CD8^+^T-cells** | | | | | | | | | | | | | | | |
|  |  | **HC** | |  | **RRMS** | |  | **SPMS** | |  | **PPMS** | |  |  |  |  |  |  | **HC** | |  | **RRMS** | |  | **SPMS** | |  | **PPMS** | |  |  |  |  |
|  |  | Mean | SE |  | Mean | SE |  | Mean | SE |  | Mean | SE |  | ANOVA/ ANCOVA | q-value (FDR) | Corr. with age |  |  | Mean | SE |  | Mean | SE |  | Mean | SE |  | Mean | SE |  | ANOVA/ ANCOVA | q-value (FDR) | Corr. with age |
|  | Cells/µl | 1052 | 73 |  | 1135 | 86 |  | 1016 | 98 |  | 1031 | 101 |  | 0.18 | 0.27 | **-0.44*** |  | Cells/µl | 517 | 47 |  | 462 | 42 |  | 495 | 79 |  | 363 | 33 |  | 0.14 | 0.25 | NS |
|  | % of T-cells | 62.9 | 1.3 |  | 67.0 | 1.9 |  | 63.7 | 2.7 |  | 69.8 | 2.0 |  | **0.04** | 0.12 | NS |  | % of T-cells | 30.2 | 1.2 |  | 27.8 | 1.6 |  | 31.2 | 2.6 |  | 24.0 | 1.7 |  | **0.03** | 0.11 | NS |
|  |  |  |  |  |  |  |  |  |  |  |  |  |  |  |  |  |  |  |  |  |  |  |  |  |  |  |  |  |  |  |  |  |  |
| T_Reg_ | CD4+T_Reg_% | 8.0 | 0.3 |  | 8.0 | 0.5 |  | 8.4 | 0.4 |  | 8.1 | 0.5 |  | 0.89 | 0.58 | NS |  |  |  |  |  |  |  |  |  |  |  |  |  |  |  |  |  |
|  | CD31+% of CD4+T_Reg_ | 21.5 | 1.2 |  | 22.1 | 1.4 |  | **12.3⁰⁰** | 1.1 |  | **16.7⁰** | 1.7 |  | **0.000003** | **0.0002** | NS |  |  |  |  |  |  |  |  |  |  |  |  |  |  |  |  |  |
|  |  |  |  |  |  |  |  |  |  |  |  |  |  |  |  |  |  |  |  |  |  |  |  |  |  |  |  |  |  |  |  |  |  |
| Th1 | IL12RB2+% | 0.8 | 0.1 |  | 0.8 | 0.1 |  | 1.0 | 0.1 |  | 0.9 | 0.1 |  | 0.14 | 0.24 | NS |  | IL12RB2+% | 0.7 | 0.1 |  | 0.6 | 0.1 |  | 0.7 | 0.1 |  | 0.8 | 0.1 |  | 0.38 | 0.39 | **0.46**** |
|  | CXCR3+% | 30.3 | 1.6 |  | 24.1 | 1.9 |  | 24.0 | 1.8 |  | 25.3 | 2.3 |  | **0.04** | 0.11 | NS |  | CXCR3+% | 50.4 | 2.0 |  | 47.9 | 2.6 |  | 40.4 | 3.2 |  | 43.0 | 3.1 |  | **0.03** | 0.11 | NS |
|  |  |  |  |  |  |  |  |  |  |  |  |  |  |  |  |  |  |  |  |  |  |  |  |  |  |  |  |  |  |  |  |  |  |
| Th17 | CCR6+% | 23.3 | 1.5 |  | 20.8 | 1.4 |  | 23.9 | 1.9 |  | 24.5 | 2.2 |  | 0.52 | 0.48 | NS |  | CCR6+% | 11.5 | 1.3 |  | 9.2 | 1.4 |  | 9.4 | 1.3 |  | 11.4 | 1.2 |  | 0.46 | 0.46 | NS |
|  | IL23R+% | 0.4 | 0.03 |  | 0.6 | 0.1 |  | **0.8⁰⁰** | 0.1 |  | **0.7⁰⁰** | 0.1 |  | **0.00001** | **0.0004** | NS |  | IL23R+% | 0.5 | 0.1 |  | 0.9 | 0.2 |  | 0.6 | 0.1 |  | 0.6 | 0.1 |  | 0.14 | 0.24 | NS |
|  |  |  |  |  |  |  |  |  |  |  |  |  |  |  |  |  |  |  |  |  |  |  |  |  |  |  |  |  |  |  |  |  |  |
| T_FH_ | CXCR5+% | 10.5 | 0.6 |  | 10.5 | 0.8 |  | 10.4 | 0.7 |  | 9.3 | 0.7 |  | 0.56 | 0.51 | NS |  | CXCR5+% | 1.9 | 0.2 |  | 2.5 | 0.3 |  | 1.9 | 0.2 |  | 2.2 | 0.4 |  | 0.36 | 0.40 | NS |
|  | Th1% of T_FH_-cells | 28.8 | 1.0 |  | **25.6⁰** | 0.8 |  | **23.5⁰⁰** | 1.3 |  | **22.1⁰⁰** | 1.3 |  | **0.0002** | **0.002** | NS |  |  |  |  |  |  |  |  |  |  |  |  |  |  |  |  |  |
|  | Th2% of T_FH_-cells | 32.2 | 1.1 |  | 34.0 | 1.9 |  | 34.7 | 1.7 |  | 34.4 | 2.1 |  | 0.62 | 0.52 | NS |  |  |  |  |  |  |  |  |  |  |  |  |  |  |  |  |  |
|  | Th17% of T_FH_-cells | 28.3 | 1.0 |  | 30.2 | 1.4 |  | 32.3 | 1.6 |  | **34.1†** | 1.0 |  | **0.002** | **0.018** | **0.44*** |  |  |  |  |  |  |  |  |  |  |  |  |  |  |  |  |  |
|  | Th17+Th2/Th1 T_FH_-cells | 2.2 | 0.1 |  | 2.6 | 0.1 |  | **3.1⁰⁰** | 0.2 |  | **3.5⁰⁰** | 0.4 |  | **0.0006** | **0.007** | NS |  |  |  |  |  |  |  |  |  |  |  |  |  |  |  |  |  |
|  | ICOS+% of T_FH_-cells | 1.1 | 0.1 |  | **2.2⁰** | 0.5 |  | **1.7⁰⁰** | 0.2 |  | 1.2 | 0.1 |  | **0.002** | **0.02** | NS |  |  |  |  |  |  |  |  |  |  |  |  |  |  |  |  |  |
|  | PD1+% of T_FH_-cells | 23.5 | 1.4 |  | 20.3 | 1.7 |  | 23.2 | 1.7 |  | 19.2 | 1.4 |  | 0.15 | 0.24 | NS |  |  |  |  |  |  |  |  |  |  |  |  |  |  |  |  |  |
|  | CD40L+% of T_FH_-cells | 0.9 | 0.1 |  | 1.4 | 0.3 |  | 0.8 | 0.2 |  | 1.0 | 0.1 |  | 0.07 | 0.17 | NS |  |  |  |  |  |  |  |  |  |  |  |  |  |  |  |  |  |
|  | OX40+% of T_FH_-cells | 3.5 | 0.5 |  | 4.5 | 0.9 |  | 3.4 | 0.7 |  | 3.6 | 0.6 |  | 0.62 | 0.51 | NS |  |  |  |  |  |  |  |  |  |  |  |  |  |  |  |  |  |
|  |  |  |  |  |  |  |  |  |  |  |  |  |  |  |  |  |  |  |  |  |  |  |  |  |  |  |  |  |  |  |  |  |  |
|  | CD31+% | 32.0 | 1.7 |  | 35.5 | 2.0 |  | **21.5⁰⁰** | 1.7 |  | **25.8⁰** | 2.1 |  | **0.00001** | **0.0003** | NS |  | CD31+% | 74.2 | 1.8 |  | 77.5 | 2.6 |  | 66.0 | 3.0 |  | 68.4 | 2.8 |  | **0.0001** | **0.002** | **-0.48**** |
|  | CD161+% | 10.1 | 0.7 |  | 9.5 | 0.9 |  | 9.9 | 1.1 |  | 11.6 | 1.0 |  | 0.46 | 0.46 | NS |  | CD161+% | 13.7 | 1.3 |  | 11.6 | 1.5 |  | 11.2 | 1.4 |  | 13.6 | 1.6 |  | 0.50 | 0.47 | NS |
|  | CCR5+% | 0.5 | 0.1 |  | 0.5 | 0.1 |  | 0.6 | 0.1 |  | 0.6 | 0.1 |  | 0.75 | 0.55 | NS |  | CCR5+% | 1.7 | 0.5 |  | 1.0 | 0.3 |  | 0.6 | 0.1 |  | 0.7 | 0.3 |  | 0.16 | 0.25 | NS |
|  | CCR2+% | 0.1 | 0.01 |  | 0.2 | 0.03 |  | 0.2 | 0.02 |  | 0.2 | 0.05 |  | 0.08 | 0.18 | NS |  | CCR2+% | 0.1 | 0.01 |  | 0.1 | 0.02 |  | 0.1 | 0.01 |  | 0.2 | 0.05 |  | **0.02** | 0.07 | NS |
|  | ICOS+% | 0.7 | 0.1 |  | 0.8 | 0.1 |  | 0.9 | 0.1 |  | 0.6 | 0.1 |  | 0.36 | 0.40 | NS |  | ICOS+% | 0.3 | 0.03 |  | **0.44⁰** | 0.1 |  | **0.35⁰** | 0.03 |  | 0.3 | 0.04 |  | **0.01** | **0.045** | NS |
|  | PD1+% | 12.1 | 0.9 |  | 8.5 | 1.2 |  | 11.0 | 1.2 |  | 9.3 | 1.1 |  | 0.07 | 0.18 | NS |  | PD1+% | 13.7 | 1.3 |  | 10.5 | 1.7 |  | 11.3 | 1.1 |  | 15.5 | 2.2 |  | 0.13 | 0.25 | NS |
|  | CD40L+% | 0.6 | 0.1 |  | 0.7 | 0.1 |  | 0.4 | 0.1 |  | 0.7 | 0.1 |  | 0.17 | 0.26 | NS |  | CD40L+% | 1.2 | 0.2 |  | 1.4 | 0.3 |  | 0.7 | 0.1 |  | 1.0 | 0.2 |  | 0.28 | 0.36 | **-0.37*** |
|  | OX40+% | 3.5 | 0.5 |  | 4.0 | 0.9 |  | 3.1 | 0.6 |  | 3.9 | 0.6 |  | 0.80 | 0.57 | NS |  | OX40+% | 1.7 | 0.3 |  | 2.0 | 0.5 |  | 1.6 | 0.5 |  | 2.2 | 0.5 |  | 0.72 | 0.54 | NS |

| **Table S2B** | | | | |  |  |  |  |  |  |  |  |  |  |  |  |  |  |  |  |  |  |  |  |  |  |  |  |  |  |  |  |
| --- | --- | --- | --- | --- | --- | --- | --- | --- | --- | --- | --- | --- | --- | --- | --- | --- | --- | --- | --- | --- | --- | --- | --- | --- | --- | --- | --- | --- | --- | --- | --- | --- |
|  |  |  |  |  |  |  |  |  |  |  |  |  |  |  |  |  |  |  |  |  |  |  |  |  |  |  |  |  |  |  |  |  |
| **B-cells** | | | | | | | | | | | | | | | |  | **Monocytes** | | | | | | | | | | | | | | | |
|  | **HC** | |  | **RRMS** | |  | **SPMS** | |  | **PPMS** | |  |  |  |  |  |  | **HC** | |  | **RRMS** | |  | **SPMS** | |  | **PPMS** | |  |  |  |  |
|  | Mean | SE |  | Mean | SE |  | Mean | SE |  | Mean | SE |  | ANOVA/ ANCOVA | q-value (FDR) | Corr. with age |  |  | Mean | SE |  | Mean | SE |  | Mean | SE |  | Mean | SE |  | ANOVA/ ANCOVA | q-value (FDR) | Corr. with age |
| Cells/µl | 267 | 31 |  | 256 | 29 |  | 234 | 48 |  | 211 | 23 |  | 0.75 | 0.56 | **-0.38*** |  | Cells/µl | 446 | 41 |  | 470 | 38 |  | 560 | 49 |  | 418 | 26 |  | 0.11 | 0.24 | NS |
| % of PBMCs | 7.1 | 0.4 |  | 7.1 | 0.6 |  | 5.7 | 0.7 |  | 6.5 | 0.5 |  | 0.20 | 0.29 | NS |  | % of PBMCs | 17.0 | 1.0 |  | 18.1 | 0.9 |  | **23.7⁰⁰** | 1.6 |  | 20.6 | 1.6 |  | **0.002** | **0.02** | NS |
|  |  |  |  |  |  |  |  |  |  |  |  |  |  |  |  |  |  |  |  |  |  |  |  |  |  |  |  |  |  |  |  |  |
| DCSIGN+% | 2.1 | 0.2 |  | 2.3 | 0.3 |  | **3.4‡** | 0.3 |  | 2.2 | 0.3 |  | **0.004** | **0.02** | **0.46**** |  | DCSIGN+% | 1.2 | 0.2 |  | 1.0 | 0.2 |  | 1.4 | 0.2 |  | 1.5 | 0.5 |  | 0.66 | 0.51 | NS |
| CD83+% | 2.3 | 0.2 |  | 2.9 | 0.4 |  | **3.8⁰⁰** | 0.3 |  | 2.5 | 0.3 |  | **0.003** | **0.02** | NS |  | CD83+% | 1.6 | 0.2 |  | 1.3 | 0.1 |  | 1.2 | 0.1 |  | 1.6 | 0.3 |  | 0.31 | 0.38 | NS |
| CCR7+ | 59.2 | 1.7 |  | 55.7 | 2.6 |  | 51.5 | 2.9 |  | 53.3 | 2.3 |  | 0.07 | 0.18 | NS |  | CCR7+ | 4.7 | 0.3 |  | 4.9 | 0.7 |  | 4.2 | 0.5 |  | 4.4 | 0.6 |  | 0.79 | 0.57 | NS |
| CD38+% | 75.3 | 1.7 |  | 71.8 | 3.2 |  | 73.3 | 3.3 |  | 73.0 | 2.9 |  | 0.78 | 0.57 | NS |  | CD38+% | 98.6 | 0.1 |  | 98.6 | 0.2 |  | 98.2 | 0.4 |  | 98.2 | 0.2 |  | 0.29 | 0.36 | NS |
| CD138+% | 1.5 | 0.1 |  | 1.6 | 0.2 |  | 2.3 | 0.2 |  | 1.7 | 0.2 |  | **0.01** | 0.052 | NS |  | CD138+% | 0.9 | 0.1 |  | 0.8 | 0.1 |  | 0.7 | 0.1 |  | 0.7 | 0.1 |  | 0.08 | 0.17 | NS |
| CD27+% | 39.0 | 1.7 |  | 39.2 | 2.8 |  | 37.7 | 4.3 |  | 36.4 | 3.5 |  | 0.90 | 0.59 | NS |  | CD27+% | 4.4 | 0.7 |  | 6.2 | 1.4 |  | 4.6 | 0.6 |  | 6.0 | 1.5 |  | 0.50 | 0.46 | NS |
| Plasmablasts | 2.2 | 0.3 |  | 2.7 | 0.9 |  | **2.8†** | 0.6 |  | 1.8 | 0.3 |  | **0.003** | **0.02** | **-0.41*** |  |  |  |  |  |  |  |  |  |  |  |  |  |  |  |  |  |
| CD40+% | 90.4 | 1.9 |  | 87.5 | 4.0 |  | 89.7 | 2.8 |  | 91.1 | 1.4 |  | 0.79 | 0.57 | NS |  | CD40+% | 3.5 | 1.0 |  | 1.4 | 0.3 |  | 3.8 | 1.2 |  | 1.3 | 0.4 |  | 0.09 | 0.20 | NS |
| CD70+% | 5.5 | 0.8 |  | 4.3 | 0.6 |  | 6.2 | 1.0 |  | 4.7 | 1.0 |  | 0.48 | 0.46 | NS |  | CD70+% | 0.2 | 0.02 |  | 0.2 | 0.03 |  | 0.1 | 0.01 |  | 0.2 | 0.02 |  | 0.64 | 0.51 | NS |
| CD80+% | 18.2 | 0.9 |  | 19.7 | 1.2 |  | 18.3 | 2.0 |  | 17.9 | 1.8 |  | 0.83 | 0.56 | NS |  | CD80+% | 2.3 | 0.3 |  | 2.1 | 0.3 |  | 8.1 | 5.3 |  | 2.8 | 0.4 |  | 0.25 | 0.33 | NS |
| CD86+% | 6.9 | 0.5 |  | 8.6 | 1.0 |  | 8.0 | 1.0 |  | 7.3 | 0.7 |  | 0.38 | 0.40 | NS |  | CD86+% | 46.5 | 3.7 |  | 43.5 | 4.4 |  | 45.8 | 4.8 |  | 51.6 | 4.8 |  | 0.65 | 0.51 | NS |
| ICOSL+% | 2.2 | 0.2 |  | 2.5 | 0.4 |  | 3.4 | 0.4 |  | 2.5 | 0.3 |  | 0.06 | 0.15 | **0.39*** |  | ICOSL+% | 3.0 | 0.6 |  | 3.6 | 0.7 |  | **7.3⁰** | 1.7 |  | 3.6 | 0.7 |  | **0.01** | **0.04** | NS |
| OX40L+% | 2.3 | 0.3 |  | 2.4 | 0.3 |  | 3.6 | 0.4 |  | 2.6 | 0.3 |  | **0.04** | 0.11 | NS |  | OX40L+% | 2.2 | 0.2 |  | 2.7 | 0.5 |  | 3.5 | 0.5 |  | 3.2 | 0.7 |  | 0.22 | 0.31 | NS |
| PDL1+% | 7.6 | 1.1 |  | 8.3 | 1.6 |  | 7.0 | 1.0 |  | 7.0 | 1.2 |  | 0.88 | 0.58 | NS |  | PDL1+% | 6.3 | 1.3 |  | 6.4 | 1.7 |  | 4.8 | 0.8 |  | 5.2 | 1.6 |  | 0.84 | 0.56 | NS |
| PDL2+% | 2.2 | 0.3 |  | 2.8 | 0.6 |  | 2.1 | 0.3 |  | 1.9 | 0.2 |  | 0.36 | 0.40 | NS |  | PDL2+% | 2.0 | 0.2 |  | 1.7 | 0.2 |  | 1.3 | 0.1 |  | 1.7 | 0.2 |  | 0.12 | 0.24 | NS |
| IL15+% | 1.3 | 0.1 |  | 1.6 | 0.2 |  | 1.5 | 0.1 |  | 1.4 | 0.2 |  | 0.57 | 0.50 | NS |  | IL15+% | 0.9 | 0.1 |  | 1.0 | 0.2 |  | 0.7 | 0.1 |  | 0.8 | 0.1 |  | 0.24 | 0.33 | NS |

| **Table S2C** | | | | |  |  |  |  |  |  |  |  |  |  |  |  |  |  |  |  |  |  |  |  |  |  |  |  |  |  |  |  |
| --- | --- | --- | --- | --- | --- | --- | --- | --- | --- | --- | --- | --- | --- | --- | --- | --- | --- | --- | --- | --- | --- | --- | --- | --- | --- | --- | --- | --- | --- | --- | --- | --- |
|  |  |  |  |  |  |  |  |  |  |  |  |  |  |  |  |  |  |  |  |  |  |  |  |  |  |  |  |  |  |  |  |  |
| **Myeloid dendritic cells** | | | | | | | | | | | | | | | |  | **Plasmacytoid dendritic cells** | | | | | | | | | | | | | | | |
|  | **HC** | |  | **RRMS** | |  | **SPMS** | |  | **PPMS** | |  |  |  |  |  |  | **HC** | |  | **RRMS** | |  | **SPMS** | |  | **PPMS** | |  |  |  |  |
|  | Mean | SE |  | Mean | SE |  | Mean | SE |  | Mean | SE |  | ANOVA/ ANCOVA | q-value (FDR) | Corr. with age |  |  | Mean | SE |  | Mean | SE |  | Mean | SE |  | Mean | SE |  | ANOVA/ ANCOVA | q-value (FDR) | Corr. with age |
| % of PBMCs | 0.5 | 0.02 |  | 0.6 | 0.1 |  | 0.6 | 0.04 |  | 0.6 | 0.04 |  | 0.61 | 0.52 | NS |  | % of PBMCs | 0.5 | 0.03 |  | 0.5 | 0.1 |  | 0.4 | 0.04 |  | 0.4 | 0.03 |  | 0.24 | 0.33 | NS |
|  |  |  |  |  |  |  |  |  |  |  |  |  |  |  |  |  |  |  |  |  |  |  |  |  |  |  |  |  |  |  |  |  |
| DCSIGN+% | 4.8 | 0.4 |  | 4.5 | 0.6 |  | 7.0 | 0.9 |  | 4.5 | 0.6 |  | **0.02** | 0.07 | NS |  | DCSIGN+% | 2.6 | 0.5 |  | 2.2 | 0.4 |  | 3.5 | 0.4 |  | 3.0 | 0.5 |  | 0.26 | 0.34 | NS |
| CD83+% | 2.5 | 0.2 |  | 2.1 | 0.2 |  | 3.0 | 0.4 |  | 2.8 | 0.5 |  | 0.21 | 0.30 | NS |  | CD83+% | 4.5 | 0.7 |  | 3.2 | 0.5 |  | 4.8 | 0.5 |  | 5.1 | 0.6 |  | 0.19 | 0.28 | NS |
| CCR7+ | 11.0 | 0.8 |  | 11.2 | 1.2 |  | 11.3 | 0.9 |  | 11.9 | 1.3 |  | 0.92 | 0.59 | NS |  | CCR7+ | 56.4 | 1.9 |  | 53.1 | 2.6 |  | 46.0 | 2.5 |  | 49.8 | 3.8 |  | **0.04** | 0.12 | NS |
| CD38+% | 98.0 | 0.2 |  | 97.6 | 0.5 |  | 97.2 | 0.9 |  | 96.8 | 0.8 |  | 0.42 | 0.43 | NS |  | CD38+% | 90.2 | 1.5 |  | 92.5 | 1.6 |  | 90.4 | 1.5 |  | 87.3 | 2.6 |  | 0.32 | 0.38 | NS |
| CD138+% | 0.7 | 0.2 |  | 0.6 | 0.1 |  | 0.5 | 0.1 |  | 0.6 | 0.1 |  | 0.96 | 0.61 | NS |  | CD138+% | 2.6 | 0.5 |  | 1.9 | 0.4 |  | 2.3 | 0.4 |  | 2.7 | 0.3 |  | 0.69 | 0.53 | NS |
| CD27+% | 14.4 | 1.8 |  | 16.3 | 1.9 |  | 18.2 | 2.9 |  | 17.3 | 2.5 |  | 0.61 | 0.51 | NS |  | CD27+% | 18.3 | 1.7 |  | 16.2 | 1.9 |  | 18.6 | 2.3 |  | 20.3 | 2.1 |  | 0.59 | 0.51 | NS |
| CD40+% | 3.0 | 0.5 |  | 1.8 | 0.4 |  | 3.4 | 0.8 |  | 2.1 | 0.2 |  | 0.14 | 0.25 | NS |  | CD40+% | 2.1 | 0.3 |  | 1.0 | 0.2 |  | 3.2 | 0.8 |  | 2.0 | 0.3 |  | **0.01** | 0.054 | NS |
| CD70+% | 0.3 | 0.1 |  | 0.2 | 0.1 |  | 0.2 | 0.1 |  | 0.2 | 0.1 |  | 0.81 | 0.57 | NS |  | CD70+% | 1.0 | 0.2 |  | 0.8 | 0.3 |  | 0.7 | 0.1 |  | 0.9 | 0.1 |  | 0.65 | 0.52 | NS |
| CD80+% | 5.5 | 0.4 |  | 5.8 | 0.6 |  | 8.4 | 1.8 |  | 6.2 | 0.6 |  | 0.13 | 0.25 | NS |  | CD80+% | 11.6 | 0.8 |  | 11.2 | 1.3 |  | 12.6 | 1.8 |  | 13.3 | 1.6 |  | 0.68 | 0.53 | NS |
| CD86+% | 37.1 | 2.1 |  | 39.3 | 1.9 |  | 37.8 | 2.6 |  | 39.5 | 2.9 |  | 0.87 | 0.58 | NS |  | CD86+% | 23.5 | 1.5 |  | 21.1 | 1.6 |  | 21.3 | 1.9 |  | 25.5 | 2.6 |  | 0.37 | 0.40 | NS |
| ICOSL+% | 2.2 | 0.2 |  | 2.4 | 0.4 |  | 2.8 | 0.5 |  | 2.2 | 0.3 |  | 0.48 | 0.46 | NS |  | ICOSL+% | 2.6 | 0.3 |  | 2.4 | 0.6 |  | 3.0 | 0.4 |  | 2.9 | 0.4 |  | 0.81 | 0.56 | NS |
| OX40L+% | 2.1 | 0.2 |  | 2.0 | 0.3 |  | 2.5 | 0.3 |  | 3.7 | 1.5 |  | 0.33 | 0.39 | NS |  | OX40L+% | 3.8 | 0.4 |  | 3.5 | 0.8 |  | 4.0 | 0.3 |  | 5.7 | 1.5 |  | 0.29 | 0.36 | NS |
| PDL1+% | 7.3 | 0.9 |  | 6.4 | 1.3 |  | 5.2 | 0.6 |  | 6.3 | 1.1 |  | 0.55 | 0.50 | NS |  | PDL1+% | 6.9 | 1.2 |  | 5.6 | 1.4 |  | 4.8 | 0.9 |  | 5.1 | 1.0 |  | 0.59 | 0.51 | NS |
| PDL2+% | 2.6 | 0.3 |  | 2.4 | 0.5 |  | 2.1 | 0.2 |  | 2.4 | 0.3 |  | 0.82 | 0.56 | NS |  | PDL2+% | 4.0 | 0.4 |  | 3.1 | 0.4 |  | 2.5 | 0.3 |  | 3.9 | 0.5 |  | **0.048** | 0.13 | NS |
| IL15+% | 1.0 | 0.3 |  | 0.8 | 0.3 |  | 0.5 | 0.1 |  | 0.8 | 0.2 |  | 0.41 | 0.42 | NS |  | IL15+% | 2.9 | 0.7 |  | 1.8 | 0.4 |  | 2.2 | 0.3 |  | 3.6 | 0.9 |  | 0.33 | 0.39 | NS |
