# Supplementary material for: Systemic Inflammation in Progressive Multiple Sclerosis Involves Follicular T-Helper, Th17- and Activated B-Cells and Correlates with Progression
Source: PLoS One. 2013 Mar 1;8(3):e57820. doi: 10.1371/journal.pone.0057820 (PMC3585852; doi:10.1371/journal.pone.0057820)
Supplement: Table S3 — Gene expression data from CD4+T–cells, CD8+T–cells, B–cells, monocytes and dendritic cells. (DOCX) [file pone.0057820.s003.docx]

**Table S3: Gene expression data from CD4^+^T-cells, CD8^+^T-cells, B-cells, monocytes and dendritic cells.** Gene expression data are shown as mean normalization ratio (NR) which reflects mRNA expression in relation to mRNA expression in a pool of peripheral mononuclear blood cells from healthy controls. (A) Gene expression in CD4^+^ and CD8^+^T-cells. (B) Gene expression in B-cells. (C) Gene expression in monocytes and dendritic cells. Statistic tests: Normally distributed parameters were analyzed by ANOVA and post-hoc testing of disease groups versus healthy controls was done with independent samples T-test. Non-normally distributed parameters were analyzed by Kruskal-Wallis test and post-hoc testing of disease groups versus healthy controls was done with Mann-Whitney test. For post-hoc tests: ^ = p≤0.05 and ^^= p≤0.01 tested by T-test and ~ = p≤0.05 and ≈ = p≤0.01 tested by Mann-Whitney. Significant values are in bold.

Abbreviations: HC = healthy controls; RRMS = relapsing-remitting multiple sclerosis in clinical remission; SPMS = secondary progressive multiple sclerosis; PPMS = primary progressive multiple sclerosis; ND = not determinable; NR= normalization ratio; T_FH_ = follicular T-helper; T_Reg_ = Regulatory T-helper cell.

| **Supplementary Table 3A** | | | | | | |  |  |  |  |  |  |  |  |  |  |  |  |  |  |  |  |  |  |  |  |  |  |  |  |  |  |  |  |  |  |
| --- | --- | --- | --- | --- | --- | --- | --- | --- | --- | --- | --- | --- | --- | --- | --- | --- | --- | --- | --- | --- | --- | --- | --- | --- | --- | --- | --- | --- | --- | --- | --- | --- | --- | --- | --- | --- |
|  |  | **CD4^+^T-cells** | | | | | | | | | | | | | | | | |  | **CD8^+^T-cells** | | | | | | | | | | | | | | | | |
|  |  | **HC** |  |  |  | **RRMS** | | |  | **SPMS** | | |  | **PPMS** | | |  |  |  | **HC** | | |  | **RRMS** | | |  | **SPMS** | | |  | **PPMS** | | |  |  |
|  | **Gene name** | N | NR Mean | SE |  | N | NR Mean | SE |  | N | NR Mean | SE |  | N | NR Mean | SE |  | Kruskal-Wallis~/ ANOVA^ |  | N | NR Mean | SE |  | N | NR Mean | SE |  | N | NR Mean | SE |  | N | NR Mean | SE |  | Kruskal-Wallis~/ ANOVA^ |
| T_Reg_ | *IL10* | 10 | 0.31 | 0.04 |  | 9 | 0.55 | 0.13 |  | 9 | 0.49 | 0.17 |  | 10 | 0.43 | 0.1 |  | 0.50^ |  | 9 | 0.53 | 0.07 |  | 10 | 0.7 | 0.13 |  | 9 | 0.35 | 0.07 |  | 9 | 0.47 | 0.21 |  | 0.31^ |
|  |  |  |  |  |  |  |  |  |  |  |  |  |  |  |  |  |  |  |  |  |  |  |  |  |  |  |  |  |  |  |  |  |  |  |  |  |
| Th1 | *IFNG* | 11 | 0.24 | 0.06 |  | 9 | 0.21 | 0.06 |  | 10 | **0.52~** | 0.18 |  | 11 | 0.16 | 0.02 |  | **0.01~** |  | 10 | 0.86 | 0.19 |  | 10 | 0.53 | 0.12 |  | 10 | 1.77 | 0.44 |  | 10 | 1.21 | 0.19 |  | **0.01^** |
|  | *TBX21* | 12 | 0.41 | 0.1 |  | 10 | 0.27 | 0.05 |  | 10 | 0.46 | 0.1 |  | 11 | 0.22 | 0.04 |  | 0.11~ |  | 10 | 1.87 | 0.59 |  | 10 | 1 | 0.22 |  | 10 | 1.97 | 0.21 |  | 10 | 1.82 | 0.36 |  | 0.26^ |
|  | *HLX* | 12 | 0.27 | 0.06 |  | 10 | 0.49 | 0.18 |  | 10 | 0.33 | 0.12 |  | 11 | 0.4 | 0.11 |  | 0.60~ |  | 10 | 0.04 | 0.02 |  | 10 | 0.06 | 0.03 |  | 10 | **0.16≈** | 0.06 |  | 9 | 0.19 | 0.13 |  | **0.046~** |
|  |  |  |  |  |  |  |  |  |  |  |  |  |  |  |  |  |  |  |  |  |  |  |  |  |  |  |  |  |  |  |  |  |  |  |  |  |
| Th2 | *IL4* | 5 | 0.18 | 0.12 |  | 4 | 0.04 | 0.01 |  | 6 | 0.08 | 0.02 |  | 7 | 0.07 | 0.02 |  | 0.47~ |  | 9 | 0.21 | 0.07 |  | 4 | 0.06 | 0.02 |  | 8 | 0.27 | 0.08 |  | 9 | 0.15 | 0.03 |  | 0.28^ |
|  | *GATA3* | 12 | 5.25 | 0.56 |  | 10 | 5.51 | 0.59 |  | 10 | 5.03 | 0.38 |  | 11 | 4.87 | 0.34 |  | 0.82^ |  | 10 | 3.31 | 0.46 |  | 10 | 3.12 | 0.48 |  | 10 | **6.00^^** | 0.76 |  | 10 | **5.94^** | 1.02 |  | **0.005^** |
|  |  |  |  |  |  |  |  |  |  |  |  |  |  |  |  |  |  |  |  |  |  |  |  |  |  |  |  |  |  |  |  |  |  |  |  |  |
| Th3 | *TGFB1* | 12 | 1.06 | 0.11 |  | 10 | 1.18 | 0.15 |  | 10 | 1.25 | 0.08 |  | 11 | 1.19 | 0.12 |  | 0.70^ |  | 10 | 1.2 | 0.16 |  | 10 | 1.06 | 0.15 |  | 10 | 1.54 | 0.09 |  | 10 | **1.72^** | 0.17 |  | **0.01^** |
|  |  |  |  |  |  |  |  |  |  |  |  |  |  |  |  |  |  |  |  |  |  |  |  |  |  |  |  |  |  |  |  |  |  |  |  |  |
| Th17 | *IL17A* | 2 | 1.77 | 0.1 |  | 2 | 1.16 | 0.48 |  | 3 | 3.52 | 1.88 |  | 3 | 1.28 | 0.32 |  | 0.58^ |  |  | ND |  |  |  | ND |  |  |  | ND |  |  |  | ND |  |  |  |
|  | *IL23R* | 10 | 0.72 | 0.2 |  | 8 | 0.52 | 0.12 |  | 9 | 0.65 | 0.11 |  | 11 | 0.61 | 0.13 |  | 0.84^ |  | 10 | 0.54 | 0.19 |  | 10 | 0.74 | 0.13 |  | 10 | 0.58 | 0.1 |  | 9 | 0.47 | 0.12 |  | 0.56^ |
|  | *RORC* | 12 | 2.85 | 0.51 |  | 10 | 2.18 | 0.26 |  | 10 | 2.91 | 0.41 |  | 11 | 2.27 | 0.26 |  | 0.43^ |  | 10 | 0.89 | 0.29 |  | 10 | 0.97 | 0.19 |  | 10 | 1.46 | 0.22 |  | 10 | 1.3 | 0.21 |  | 0.29^ |
|  |  |  |  |  |  |  |  |  |  |  |  |  |  |  |  |  |  |  |  |  |  |  |  |  |  |  |  |  |  |  |  |  |  |  |  |  |
| T_FH_ | *ICOS* | 12 | 1.87 | 0.15 |  | 10 | 2 | 0.15 |  | 10 | **2.46^** | 0.21 |  | 11 | **2.33^** | 0.14 |  | **0.04^** |  | 10 | 1.04 | 0.09 |  | 10 | 0.93 | 0.05 |  | 10 | 1.21 | 0.08 |  | 10 | 1.04 | 0.08 |  | 0.09^ |
|  | *IL21* | 12 | 1.49 | 0.42 |  | 10 | 1.12 | 0.23 |  | 10 | **4.21~** | 2.09 |  | 11 | 1.25 | 0.2 |  | **0.02~** |  | 3 | 0.33 | 0.07 |  | 1 | 0.37 | 0 |  | 4 | 0.32 | 0.05 |  | 4 | 0.38 | 0.12 |  | 0.96^ |
|  | *IL21R* | 12 | 2.09 | 0.23 |  | 10 | 2.39 | 0.14 |  | 10 | **2.88^** | 0.16 |  | 11 | **2.86^** | 0.16 |  | **0.008^** |  | 10 | 2.66 | 0.35 |  | 10 | 2.36 | 0.31 |  | 10 | 2.61 | 0.18 |  | 10 | 2.54 | 0.27 |  | 0.88^ |
|  |  |  |  |  |  |  |  |  |  |  |  |  |  |  |  |  |  |  |  |  |  |  |  |  |  |  |  |  |  |  |  |  |  |  |  |  |
|  | *TNFA* | 12 | 0.95 | 0.12 |  | 10 | 0.77 | 0.07 |  | 10 | 1.23 | 0.08 |  | 11 | 1.09 | 0.08 |  | **0.01^** |  | 10 | 0.52 | 0.14 |  | 10 | 0.3 | 0.06 |  | 10 | **1.04^** | 0.13 |  | 9 | 0.86 | 0.13 |  | **0.0003^** |
|  | *LTA* | 11 | 1.82 | 0.16 |  | 10 | 2.04 | 0.2 |  | 10 | 2.27 | 0.21 |  | 11 | 2.4 | 0.17 |  | 0.13^ |  | 10 | 1.55 | 0.15 |  | 10 | 1.72 | 0.16 |  | 10 | 1.87 | 0.11 |  | 10 | 1.8 | 0.12 |  | 0.41^ |
|  | *LTB* | 12 | 4.87 | 0.58 |  | 10 | 5.76 | 0.74 |  | 10 | **7.17^** | 0.61 |  | 11 | **7.88^^** | 0.42 |  | **0.003^** |  | 10 | 2.16 | 0.36 |  | 10 | 2.69 | 0.33 |  | 10 | **3.81^^** | 0.27 |  | 10 | **4.21^^** | 0.43 |  | **0.0006^** |
|  | *LTBR* | 11 | 0.05 | 0.01 |  | 10 | 0.05 | 0.01 |  | 10 | 0.06 | 0.02 |  | 11 | 0.07 | 0.02 |  | 0.59^ |  | 9 | 0.01 | 0 |  | 7 | 0.01 | 0 |  | 10 | **0.09≈** | 0.05 |  | 10 | **0.04~** | 0.02 |  | **0.006~** |
|  | *TNFSF14* | 11 | 0.73 | 0.14 |  | 10 | 0.59 | 0.07 |  | 10 | 1.01 | 0.09 |  | 11 | 0.73 | 0.08 |  | **0.04^** |  | 10 | 1.25 | 0.33 |  | 10 | 0.69 | 0.14 |  | 10 | **2.12^** | 0.25 |  | 10 | 1.93 | 0.37 |  | **0.003^** |
|  | *TNFRSF14* | 12 | 1.23 | 0.16 |  | 10 | 1.28 | 0.14 |  | 10 | 1.18 | 0.05 |  | 11 | 1.18 | 0.05 |  | 0.47~ |  | 10 | 1.04 | 0.16 |  | 10 | 1.06 | 0.22 |  | 10 | 1.2 | 0.05 |  | 10 | 1.17 | 0.07 |  | 0.83^ |

| **Supplementary Table 3B** | | | | | | |  |  |  |  |  |  |  |  |  |  |  |
| --- | --- | --- | --- | --- | --- | --- | --- | --- | --- | --- | --- | --- | --- | --- | --- | --- | --- |
|  | **B-cells** | | | | | | | | | | | | | | | | |
|  | **HC** | | |  | **RRMS** | | |  | **SPMS** | | |  | **PPMS** | | |  |  |
| **Gene name** | N | NR Mean | SE |  | N | NR Mean | SE |  | N | NR Mean | SE |  | N | NR Mean | SE |  | Kruskal-Wallis~/ ANOVA^ |
| *IL10* | 3 | 0.08 | 0.03 |  | 6 | 0.21 | 0.04 |  | 4 | 0.2 | 0.08 |  | 6 | 0.16 | 0.06 |  | 0.52^ |
|  |  |  |  |  |  |  |  |  |  |  |  |  |  |  |  |  |  |
| *IFNG* | 2 | 0.03 | 0.01 |  | 1 | 0.06 | 0 |  | 3 | 0.04 | 0.02 |  | 1 | 0.06 | 0 |  | 0.79^ |
|  |  |  |  |  |  |  |  |  |  |  |  |  |  |  |  |  |  |
| *IL1B* | 7 | 0.17 | 0.04 |  | 7 | 0.34 | 0.09 |  | 9 | 0.45 | 0.19 |  | 11 | 0.42 | 0.19 |  | 0.57~ |
| *IL6* | 8 | 10.47 | 1.07 |  | 9 | 14.47 | 2.22 |  | 9 | 9.64 | 1.19 |  | 11 | 14.45 | 2.42 |  | 0.16^ |
| *IL12A* | 8 | 3.22 | 0.47 |  | 9 | 3.25 | 0.37 |  | 9 | 4.13 | 0.53 |  | 11 | 4.11 | 0.28 |  | 0.22^ |
| *IL12B* | 4 | 11.42 | 2.85 |  | 4 | 16.18 | 5.26 |  | 8 | 16.1 | 5.1 |  | 7 | 11.66 | 2.01 |  | 0.79^ |
| *IL23* | 8 | 0.82 | 0.09 |  | 9 | 0.78 | 0.19 |  | 9 | 0.49 | 0.08 |  | 10 | 0.55 | 0.03 |  | 0.13^ |
|  |  |  |  |  |  |  |  |  |  |  |  |  |  |  |  |  |  |
| *TNFA* | 8 | 0.71 | 0.1 |  | 8 | 0.68 | 0.06 |  | 9 | 0.97 | 0.14 |  | 11 | 1.02 | 0.09 |  | 0.051^ |
| *LTA* | 8 | 0.89 | 0.08 |  | 9 | 0.94 | 0.1 |  | 9 | **1.36^** | 0.14 |  | 11 | 1.43 | 0.23 |  | **0.048^** |
| *LTB* | 8 | 7.78 | 0.57 |  | 9 | 6.69 | 1.23 |  | 10 | 6.19 | 0.64 |  | 11 | 6.49 | 0.35 |  | 0.49^ |
| *LTBR* | 8 | 0.06 | 0.02 |  | 7 | 0.05 | 0.01 |  | 9 | 0.21 | 0.08 |  | 11 | 0.14 | 0.04 |  | **0.046~** |
| *TNFRSF14* | 8 | 0.98 | 0.08 |  | 9 | 0.97 | 0.13 |  | 10 | 1.09 | 0.07 |  | 11 | 1.1 | 0.04 |  | 0.54^ |
| *TNFSF14* | 4 | 0.14 | 0.07 |  | 5 | 0.04 | 0.02 |  | 7 | 0.12 | 0.05 |  | 9 | 0.08 | 0.03 |  | 0.46^ |
|  |  |  |  |  |  |  |  |  |  |  |  |  |  |  |  |  |  |
| *IL21R* | 8 | 2.09 | 0.19 |  | 9 | 2.07 | 0.32 |  | 9 | 2.37 | 0.33 |  | 11 | 1.85 | 0.21 |  | 0.58^ |
| *IGJ* | 9 | 9.43 | 1.98 |  | 8 | 6.07 | 1.12 |  | 10 | 10.94 | 1.87 |  | 11 | 9.5 | 1.68 |  | 0.29^ |

| **Supplementary Table 3C** | | | | | | |  |  |  |  |  |  |  |  |  |  |  |  |  |  |  |  |  |  |  |  |  |  |  |  |  |  |  |  |  |
| --- | --- | --- | --- | --- | --- | --- | --- | --- | --- | --- | --- | --- | --- | --- | --- | --- | --- | --- | --- | --- | --- | --- | --- | --- | --- | --- | --- | --- | --- | --- | --- | --- | --- | --- | --- |
|  | **Monocytes** | | | | | | | | | | | | | | |  |  |  | **Dendritic cells** | | | | | | | | | | | | | | | | |
|  | **HC** | | |  | **RRMS** | | |  | **SPMS** | | |  | **PPMS** | | |  |  |  | **HC** | | |  | **RRMS** | | |  | **SPMS** | | |  | **PPMS** | | |  |  |
| **Gene name** | N | NR Mean | SE |  | N | NR Mean | SE |  | N | NR Mean | SE |  | N | NR Mean | SE |  | Kruskal-Wallis~/ ANOVA^ |  | N | NR Mean | SE |  | N | NR Mean | SE |  | N | NR Mean | SE |  | N | NR Mean | SE |  | Kruskal-Wallis~/ ANOVA^ |
| *IL10* | 10 | 1.22 | 0.18 |  | 11 | 1.52 | 0.17 |  | 9 | 1.73 | 0.34 |  | 8 | 1.41 | 0.24 |  | 0.47^ |  | 1 | 0.34 | 0 |  | 5 | 0.16 | 0.02 |  | 1 | 0.29 | 0 |  | 5 | 0.27 | 0.13 |  | 0.77^ |
|  |  |  |  |  |  |  |  |  |  |  |  |  |  |  |  |  |  |  |  |  |  |  |  |  |  |  |  |  |  |  |  |  |  |  |  |
| *IL1B* | 11 | 8.34 | 1.33 |  | 11 | 23.2 | 7.83 |  | 9 | 14.3 | 6.42 |  | 8 | 13.9 | 7.3 |  | 0.43~ |  | 12 | 4.07 | 0.5 |  | 10 | 4.71 | 0.8 |  | 8 | 2.97 | 0.6 |  | 11 | 4.68 | 1.11 |  | 0.47^ |
| *IL6* | 8 | 0.29 | 0.08 |  | 8 | 0.63 | 0.09 |  | 9 | 0.87 | 0.61 |  | 6 | 1.82 | 1.61 |  | 0.06~ |  | 6 | 0.29 | 0.1 |  | 5 | 0.34 | 0.1 |  | 2 | 0.8 | 0.13 |  | 8 | 0.68 | 0.14 |  | 0.09^ |
| *IL12A* | 11 | 0.53 | 0.09 |  | 11 | 0.41 | 0.04 |  | 9 | 0.43 | 0.05 |  | 8 | 0.45 | 0.05 |  | 0.51^ |  | 8 | 0.11 | 0.03 |  | 4 | 0.16 | 0.02 |  | 5 | 0.17 | 0.06 |  | 6 | 0.24 | 0.03 |  | 0.11^ |
| *IL12B* |  | ND |  |  |  | ND |  |  |  | ND |  |  |  | ND |  |  |  |  |  | ND |  |  |  | ND |  |  |  | ND |  |  |  | ND |  |  |  |
| *IL23* | 9 | 0.08 | 0.01 |  | 8 | **0.05^** | 0.01 |  | 7 | **0.05^** | 0 |  | 8 | **0.04^** | 0.01 |  | **0.01^** |  | 6 | 0.03 | 0 |  | 9 | 0.04 | 0.01 |  | 6 | 0.02 | 0 |  | 9 | 0.05 | 0.01 |  | 0.13^ |
|  |  |  |  |  |  |  |  |  |  |  |  |  |  |  |  |  |  |  |  |  |  |  |  |  |  |  |  |  |  |  |  |  |  |  |  |
| *TNFA* | 11 | 2.15 | 0.35 |  | 11 | 2.11 | 0.31 |  | 9 | 2.92 | 0.71 |  | 8 | 2.57 | 0.48 |  | 0.56^ |  | 12 | 0.55 | 0.05 |  | 10 | 0.62 | 0.07 |  | 7 | 0.67 | 0.17 |  | 11 | 0.88 | 0.14 |  | 0.14^ |
| *LTA* | 11 | 0.16 | 0.02 |  | 9 | 0.1 | 0.02 |  | 9 | 0.16 | 0.02 |  | 7 | 0.15 | 0.02 |  | 0.27^ |  | 11 | 0.27 | 0.05 |  | 8 | 0.36 | 0.06 |  | 6 | 0.32 | 0.07 |  | 10 | 0.31 | 0.05 |  | 0.65^ |
| *LTB* | 11 | 0.27 | 0.05 |  | 11 | **0.12^** | 0.03 |  | 9 | 0.21 | 0.02 |  | 8 | 0.25 | 0.04 |  | **0.046^** |  | 12 | 1.43 | 0.2 |  | 10 | 1.55 | 0.2 |  | 8 | 3.07 | 0.76 |  | 11 | 1.96 | 0.3 |  | 0.07~ |
| *LTBR* | 11 | 3.81 | 0.44 |  | 11 | 3.19 | 0.34 |  | 9 | **6.34^^** | 0.35 |  | 8 | **7.09^^** | 0.58 |  | **0.0000001^** |  | 12 | 1.75 | 0.2 |  | 10 | 1.72 | 0.12 |  | 7 | 2.28 | 0.17 |  | 11 | **3.07^^** | 0.18 |  | **0.000005^** |
| *TNFSF14* | 10 | 1.33 | 0.22 |  | 11 | 0.79 | 0.16 |  | 8 | 1.61 | 0.24 |  | 8 | 1.34 | 0.17 |  | **0.03^** |  | 12 | 0.13 | 0.02 |  | 9 | 0.17 | 0.03 |  | 6 | 0.17 | 0.04 |  | 10 | 0.2 | 0.03 |  | 0.34^ |
| *TNFRSF14* | 11 | 1.27 | 0.28 |  | 11 | 1.04 | 0.13 |  | 8 | **1.43~** | 0.07 |  | 8 | **1.47≈** | 0.04 |  | **0.003~** |  | 12 | 0.66 | 0.09 |  | 10 | 0.65 | 0.03 |  | 6 | 0.7 | 0.06 |  | 11 | 0.75 | 0.02 |  | 0.62^ |
|  |  |  |  |  |  |  |  |  |  |  |  |  |  |  |  |  |  |  |  |  |  |  |  |  |  |  |  |  |  |  |  |  |  |  |  |
| *IL21R* | 10 | 0.37 | 0.06 |  | 11 | **0.18^** | 0.04 |  | 9 | 0.39 | 0.1 |  | 7 | 0.53 | 0.12 |  | **0.03^** |  | 12 | 0.39 | 0.03 |  | 10 | 0.33 | 0.04 |  | 7 | 0.56 | 0.1 |  | 11 | **0.66^^** | 0.08 |  | **0.002^** |
