# Supplementary figures and images for: Systemic Inflammation in Progressive Multiple Sclerosis Involves Follicular T-Helper, Th17- and Activated B-Cells and Correlates with Progression
Source: PLoS One. 2013 Mar 1;8(3):e57820. doi: 10.1371/journal.pone.0057820 (PMC3585852; doi:10.1371/journal.pone.0057820)

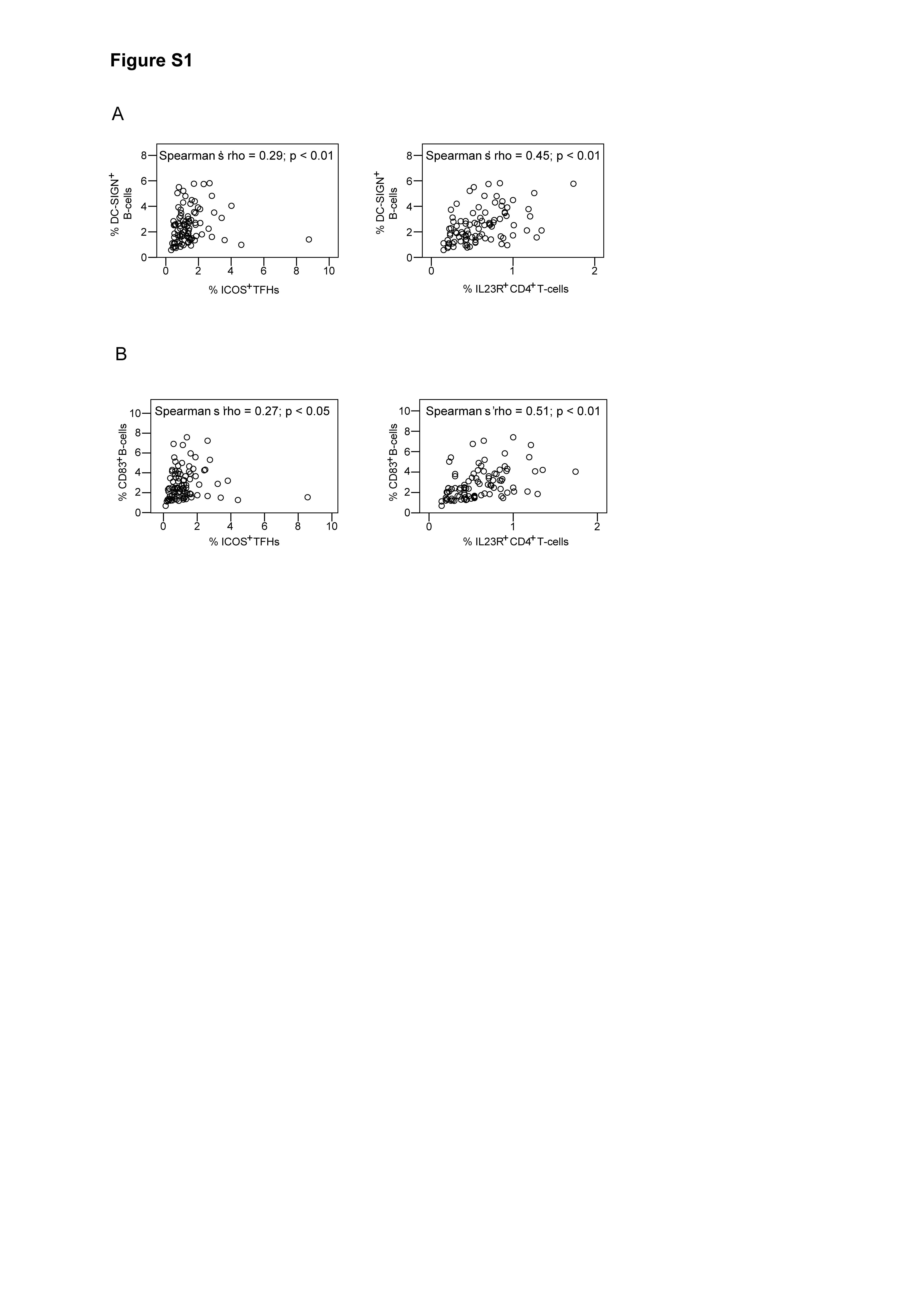

Supplement: Figure S1 — (TIF) [file pone.0057820.s004.tif]
